# Targeting the Oral Mucosa: Emerging Drug Delivery Platforms and the Therapeutic Potential of Glycosaminoglycans

**DOI:** 10.3390/pharmaceutics17091212

**Published:** 2025-09-17

**Authors:** Bruno Špiljak, Maja Somogyi Škoc, Iva Rezić Meštrović, Krešimir Bašić, Iva Bando, Ivana Šutej

**Affiliations:** 1 Department of Oral Medicine, University of Zagreb School of Dental Medicine, 10000 Zagreb, Croatia; 2 Faculty of Textile Technology, University of Zagreb, 10000 Zagreb, Croatia; maja.somogyi@ttf.unizg.hr (M.S.Š.); iva.rezic@ttf.unizg.hr (I.R.M.); 3 Department of Pharmacology, University of Zagreb School of Dental Medicine, 10000 Zagreb, Croatia; basic@sfzg.hr; 4 Dental Polyclinic Zagreb, 10000 Zagreb, Croatia; ibando1@sfzg.hr; 5 University of Zagreb School of Dental Medicine, 10000 Zagreb, Croatia

**Keywords:** oral mucosa, drug delivery systems, glycosaminoglycans, bioavailability, controlled release

## Abstract

Research into oral mucosa-targeted drug delivery systems (DDS) is rapidly evolving, with growing emphasis on enhancing bioavailability and precision targeting while overcoming the unique anatomical and physiological barriers of the oral environment. Despite considerable progress, challenges such as enzymatic degradation, limited mucosal penetration, and solubility issues continue to hinder therapeutic success. Recent advancements have focused on innovative formulation strategies—including nanoparticulate and biomimetic systems—to improve delivery efficiency and systemic absorption. Simultaneously, smart and stimuli-responsive materials are emerging, offering dynamic, environment-sensitive drug release profiles. One particularly promising area involves the application of glycosaminoglycans, a class of naturally derived polysaccharides with excellent biocompatibility, mucoadhesive properties, and hydrogel-forming capacity. These materials not only enhance drug residence time at the mucosal site but also enable controlled release kinetics, thereby improving therapeutic outcomes. However, critical research gaps remain: standardized, clinically meaningful mucoadhesion/permeation assays and robust in vitro–in vivo correlations are still lacking; long-term stability, batch consistency of GAGs, and clear regulatory classification (drug, device, or combination) continue to impede scale-up and translation. Patient-centric performance—palatability, mouthfeel, discreet wearability—and head-to-head trials versus standard care also require systematic evaluation to guide adoption. Overall, converging advances in GAG-based films, hydrogels, and nanoengineered carriers position oral mucosal delivery as a realistic near-term option for precision local and selected systemic therapies—provided the field resolves standardization, stability, regulatory, and usability hurdles.

## 1. Introduction

The oral mucosa is increasingly recognized as a promising site for both local and systemic drug delivery. Its rich vascularization, relative permeability, and ease of access make it a compelling alternative to conventional routes such as oral ingestion or intravenous administration, especially for drugs that are susceptible to degradation in the gastrointestinal tract or extensive hepatic first-pass metabolism [1,2,3,4,5]. Furthermore, oral mucosal delivery offers advantages such as avoidance of gastrointestinal irritation, ease of administration, and better patient compliance, particularly in populations with swallowing difficulties or chronic conditions [6,7,8].

The oral cavity encompasses multiple distinct regions, each with different anatomical and physiological characteristics that influence drug absorption. These include the sublingual, buccal, gingival, and palatal mucosae. Among these, the buccal and sublingual regions are most commonly utilized for drug delivery due to their favorable permeability and accessibility [2,9,10,11,12]. However, despite these advantages, the route is not without challenges. Salivary washout, enzymatic degradation, and limited surface area for adhesion all contribute to reduced drug retention and bioavailability [2,13,14].

Glycosaminoglycans (GAGs) are linear polysaccharides composed of repeating disaccharides (an amino sugar with a uronic acid or galactose) and are classified into sulfated families—chondroitin/dermatan sulfate, heparin/heparan sulfate, keratan sulfate—and the non-sulfated hyaluronan. Their high anionic charge and polydispersity impart strong hydration, viscoelasticity, and wide protein-binding capacity [15,16,17]. These physicochemical features are directly relevant to oromucosal delivery: hydrated interfacial layers support lubrication and mucoadhesion, while sequence- and position-specific sulfation motifs (“sulfation code”) govern selective interactions with cytokines and growth factors, enabling bioactive matrices [14,18,19]. Hyaluronan further engages the CD44 receptor, widely expressed and often upregulated in inflamed or malignant oral tissues, providing a basis for tissue retention and receptor-mediated cell interactions exploitable in targeted formulations [20,21,22]. The oral environment also contains native hyaluronan and hyaluronidase activity, highlighting the need to tune molecular weight, crosslinking, and formulation context to balance residence time with controlled biodegradation at the mucosal surface [16,17,23]. Together, these structure–property relationships justify a focused appraisal of GAG-based films, hydrogels, and nanoengineered carriers as modular materials for oromucosal therapy, while clarifying where enzymatic turnover and receptor biology can be leveraged—or must be mitigated—in translational design [21,22,24]. GAGs offer clear advantages for oromucosal delivery—biocompatibility, intrinsic mucoadhesion, tunable charge/hydration, and, in the case of hyaluronan, receptor-mediated interactions that can improve localization and retention [16,17]. These benefits are counterweighted by practical limitations, including batch-to-batch variability of natural sources, enzymatic degradation in saliva, challenges in maintaining mechanical integrity and palatability during wear, and incomplete standardization of mucoadhesion/permeation assays [6,25,26]. Translation is further constrained by stability and sterilization requirements [19,27,28], ambiguity around drug–device–combination classification [29,30,31,32], and a scarcity of head-to-head clinical data versus current standard care [33,34,35,36]. Taken together, GAG-based systems are most compelling when prolonged mucosal residence and gentle, bioactive matrices are required, but their routine adoption will depend on harmonized testing, robust life-cycle stability data, and patient-centric usability evidence. In response to these limitations, research has increasingly focused on developing advanced oral mucosal drug delivery systems (DDS) designed to prolong mucosal contact, enhance permeation, and enable controlled release. Mucoadhesive polymers, nanocarriers, and stimuli-responsive hydrogels represent key innovations in this area [37,38,39,40,41,42].

Of particular interest is the incorporation of glycosaminoglycans (GAGs)—a class of naturally occurring, biocompatible polysaccharides such as chitosan, hyaluronic acid, and alginate—into DDS formulations. These biomaterials exhibit high mucoadhesiveness, hydration capacity, and modifiability, making them ideal for tailoring drug release profiles and improving therapeutic outcomes [1,2,6,43,44]. These polymers have moved from ancillary excipients to principal design elements that directly govern residence time, barrier negotiation, and on-site bioactivity in the oral cavity. Beyond generic mucoadhesion, GAGs provide tunable hydration layers, electrostatic and receptor-mediated interactions (e.g., HA–CD44), and stimuli-responsive matrices that collectively enable durable localization and controllable release in a highly dynamic, saliva-bathed environment [17,20,21,22]. Over just the past few years, their roles have expanded across clinically relevant formats—mucoadhesive films/wafers, smart hydrogels, nanoparticulate coatings, and even textile-inspired scaffolds [45,46,47,48]—bringing the field closer to translation while exposing specific standardization and regulatory gaps this review is positioned to clarify [25,26,29,30,31,32]. At the same time, comparative stability data [19,27,28], pediatric-friendly wafers [49,50,51,52], and early clinical experiences with HA-based products [45,46,47,48] underscore real-world feasibility, strengthening the case for a targeted appraisal of GAG chemistry–function relationships and platform selection for distinct oral indications. Finally, recurring bottlenecks—non-harmonized mucoadhesion/permeation assays [25,27], imperfect in vitro–in vivo correlation [26], and ambiguity around drug–device classification [29,30,31,32]—demand a consolidated, GAG-centric perspective to guide experimental design and translation.

In this review, the anatomical and physiological constraints that shape oromucosal delivery and clinical use cases are first framed. Current dosage forms and enabling technologies are then surveyed [6,53,54], to delineate where GAGs add distinctive value. Next, the major GAG families and their structure–property–performance relationships relevant to oral applications (chitosan, HA, dextran, alginate) are dissected. Building on this foundation, GAG-based platforms—mucoadhesive films/wafers, hydrogels/nanogels, nanoparticulates/microparticulates, and emerging textile scaffolds—are compared, with emphasis on formulation, stability, and therapeutic use-cases. For this review, ‘mucosal DDS’ refers primarily to retentive mucoadhesive systems engineered to remain in situ (non-disintegrating or predictably eroding) and provide unidirectional or site-focused release. Dispersive intraoral formats (e.g., orodispersible films, lozenges, sprays) are discussed only where surface coating or rapid symptom relief is the therapeutic goal. Evaluation methodologies (release, permeability, and mucoadhesion) are then examined, and areas where standardization is most urgently needed are highlighted [14,25,26]. Finally, patient-centric usability [49,50,51,52], manufacturing and regulatory considerations [29,30,31,32], and health-economic factors are discussed, and prioritized research gaps together with a translational outlook are presented.

## 2. Anatomical and Physiological Features of the Oral Mucosa Relevant to Drug Delivery

The oral cavity comprises several distinct subregions that differ in structure, function, and permeability, all of which influence the success of mucosal drug delivery. The oral mucosa is typically categorized into three types: masticatory (e.g., gingiva and hard palate), lining (e.g., buccal and sublingual areas), and specialized (e.g., dorsal tongue). Each type possesses unique histological and physiological characteristics that determine its suitability for drug absorption [14,55].

The buccal and sublingual mucosae are the most studied routes for transmucosal drug delivery. The buccal mucosa is approximately 500–800 μm thick and features a non-keratinized epithelium overlying a vascularized lamina propria, which facilitates drug permeation while providing a relatively stable environment for dosage form adherence. In contrast, the sublingual mucosa is thinner and more permeable but more prone to salivary washout, which can reduce the residence time of formulations [14,56,57].

The permeability of the mucosa is heavily influenced by the degree of keratinization, lipid content, and the organization of intercellular junctions. Non-keratinized regions allow for easier diffusion of hydrophilic molecules, whereas keratinized epithelium, with its higher lipid content and tight intercellular junctions, serves as a formidable barrier [56,58]. Moreover, the buccal mucosa is less enzymatically active than gastrointestinal tissues, reducing the degradation risk for sensitive molecules like peptides or proteins [59,60].

Saliva also plays a critical role in drug dissolution, diffusion, and metabolism. Produced at a rate of approximately 0.5–1.5 L per day, saliva contains enzymes such as amylase and esterase that can influence drug stability. Its continuous flow and varying pH (ranging from 6.2 to 7.6) can lead to dilution and removal of the drug, emphasizing the need for mucoadhesive delivery systems that can withstand such clearance [61,62,63,64].

Another emerging consideration is the interaction between drug formulations and the oral microbiome. Changes in microbial composition—whether from disease, antibiotics, or the drug carrier itself—may influence both drug efficacy and mucosal health [65,66]. Additionally, immune cells present in the mucosa, such as Langerhans cells and dendritic cells, can recognize and respond to drug components, especially in vaccine delivery or immunotherapy contexts [67,68,69].

From a histological perspective, the oral mucosa consists of a stratified squamous epithelium and a connective tissue lamina propria. While the epithelium acts as the primary barrier to drug permeation, the lamina propria is richly supplied with blood and lymphatic vessels, offering an efficient pathway for systemic absorption. The turnover rate of oral epithelial cells is also relatively rapid—about 5–10 days—which can influence both healing and absorption dynamics, particularly for chronically applied formulations [55].

Vascular drainage also plays an important role. Drugs absorbed via the sublingual and buccal mucosa primarily enter the systemic circulation through the deep lingual and facial veins, bypassing hepatic first-pass metabolism. This pharmacokinetic advantage allows for lower dosing of certain drugs and reduces the risk of hepatic toxicity. However, regional variability in vascular density and blood flow may influence interindividual differences in absorption efficiency [70,71].

Mucosal hydration is another crucial factor. Adequate hydration maintains epithelial integrity and facilitates mucoadhesion and drug diffusion [2,13]. Conditions such as xerostomia (dry mouth), common in older adults and patients undergoing chemotherapy or radiotherapy, can compromise drug delivery by altering mucosal permeability and the performance of adhesive formulations [72].

Bioadhesion refers to interfacial bonding between a material and a biological surface (cells, soft tissues, or mineralized substrates), whereas mucoadhesion denotes the mucus-specific case in which polymers adhere to the mucin layer and/or the epithelial glycocalyx of mucosal tissues. In oromucosal drug delivery, mucoadhesion is the principal design objective because it prolongs residence against salivary clearance and supports directional drug flux toward the epithelium. Mechanistically, mucoadhesion arises from a combination of wetting/adsorption, hydrogen-bonding and electrostatic interactions, and interpenetration of polymer and mucus chains; throughout this review we therefore use “mucoadhesion” unless broader bioadhesive principles are being discussed [73,74]. 

Importantly, the dynamics of oral muscle activity—including speech, chewing, and swallowing—can affect dosage form retention. These mechanical movements can displace mucoadhesive films or patches, limiting their effectiveness unless properly designed to anchor in place [6,75]. Thus, the success of mucosal delivery systems depends not only on the physicochemical compatibility between drug and tissue but also on the interplay between physiological activity and formulation robustness.

**Figure 1:**
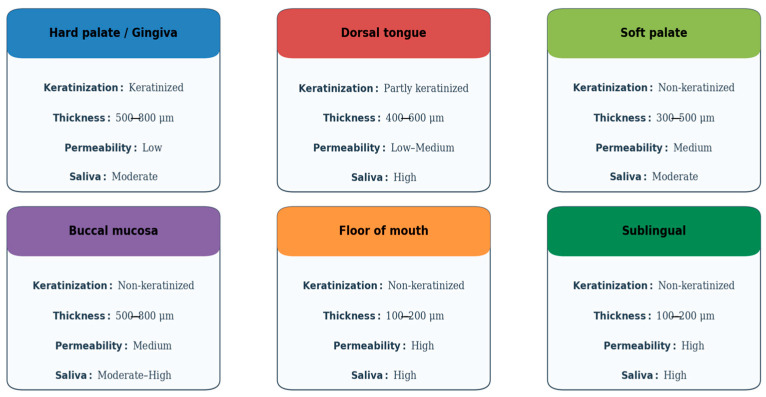
Oral mucosal subregions and salient biophysical features. Keratinization, approximate epithelial thickness, relative permeability, and salivary exposure are indicated to guide site selection and platform expectations (an original scheme based on the current literature data).

**Figure 2:**
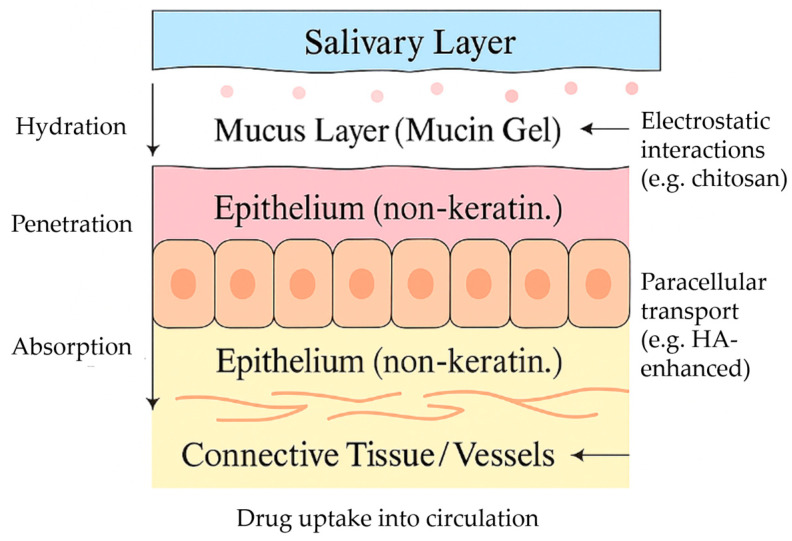
Mechanisms of mucoadhesion and drug penetration in GAG-based systems (an original scheme based on the current literature data). Mucoadhesive GAGs, such as hyaluronic acid and chitosan, interact with mucins to enhance hydration and prolong mucosal residence time. Certain GAGs also facilitate drug transport through paracellular or transcellular pathways. Additionally, GAG-based delivery platforms help protect therapeutic agents from premature degradation, enabling their absorption into systemic circulation.

As discussed above, the oral mucosa presents a complex but highly promising environment for drug delivery. A thorough understanding of its anatomical compartments, vascular and immune infrastructure, enzymatic and microbiome context, and mechanical properties is essential for designing effective therapeutic systems that maximize both local and systemic bioavailability (Figure 1). These complex anatomical and physiological factors further highlight the relevance of mucoadhesive glycosaminoglycan (GAG)-based systems, whose mechanisms of action are illustrated in Figure 2.

## 3. Barriers and Limitations in Oral Mucosal Drug Delivery

While the oral mucosa offers multiple advantages as a drug delivery site, including accessibility, non-invasiveness, and avoidance of first-pass metabolism, it also presents significant physiological and biochemical barriers that can limit the efficacy of administered therapeutics [60,76]. Understanding these limitations is crucial for designing systems that can overcome them and deliver drugs effectively.

Salivary washout represents one of the most significant challenges. The average adult produces 0.5 to 1.5 L of saliva daily, and this continuous flow can rapidly dilute or remove a drug formulation, particularly in the sublingual area. Consequently, maintaining the residence time of drug carriers long enough to allow adequate absorption becomes a major formulation goal [76,77]. Mucoadhesive systems have been developed to resist this mechanical clearance, but their effectiveness can be compromised by movement of the tongue, swallowing, and variations in salivary viscosity [78].

Enzymatic degradation is another critical barrier. Although the enzymatic activity in the oral cavity is lower than in the gastrointestinal tract, the presence of proteolytic enzymes like aminopeptidases, esterases, and lysozymes can still degrade susceptible drugs, especially peptides, nucleotides, and prodrugs. Enzyme inhibitors, protective coatings, or chemical modifications—such as polyethylene glycol (PEG)ylation—are often employed to enhance the stability of these molecules [79,80].

Disintegration of oromucosal formulations can leave insoluble or partially hydrated residues in the oral cavity—an effect reported especially for hydrocolloid-rich matrices that may not disperse completely [81]. Residual fragments and perceived stickiness are recognized determinants of end-user acceptability, contributing to discomfort, unpleasant mouthfeel, and, in some users, transient interference with speech or normal oral function [82]. Accordingly, excipient selection and dissolution kinetics should be engineered to promote complete clearance (e.g., rapidly dispersing maltodextrin-based ODFs) and verified with human acceptability panels that assess mouthfeel and ease of use alongside disintegration performance [83,84].

Backing-layer composition is a critical design variable in buccal films: impermeable layers (e.g., ethyl cellulose/Eudragit) are routinely employed to enforce unidirectional release, minimize drug loss into saliva, and direct flux toward the mucosa [85,86]. Where non-dissolvable backings are selected, the device should be removed after the intended dosing interval; alternatively, dissolvable/erodible backings may be used so that the assembly clears once drug release is complete [87]. In the absence of an appropriate backing, release occurs from both faces of the film—diluting the mucosal dose and exposing non-target tissues—whereas bilayer designs demonstrably reduce donor-side loss and improve local availability [88,89].

Permeability limitations also exist. The epithelial structure of the oral mucosa, particularly in keratinized regions such as the hard palate and gingiva, presents a strong barrier to the diffusion of hydrophilic drugs and macromolecules. The tight junctions between epithelial cells restrict paracellular transport, making transcellular permeation the primary mechanism for most compounds. This restricts the passive diffusion of large and/or polar molecules [2,14,90,91].

To enhance permeability, chemical penetration enhancers such as surfactants, bile salts, fatty acids, and cyclodextrins have been investigated. However, many of these substances are associated with cytotoxicity, irritation, or disruption of mucosal integrity, raising concerns about long-term safety and user acceptability [55,92]. Finding a balance between sufficient permeation and biocompatibility remains a key challenge in this domain.

Salivary flow varies widely (unstimulated ~0.3–0.4 mL/min; stimulated ~1.5–2.0 mL/min), and hyposalivation/xerostomia (e.g., oncology, polypharmacy) depresses flow and alters pH—both materially affect mucoadhesion, dissolution, and taste masking. Stratify in vitro tests by flow rate and pH to mimic xerostomia vs. normal [93].

Mucosal thickness/keratinization and receptor expression (e.g., CD44 in inflamed or malignant tissues) also vary across patients and lesions—report site-specific outcomes (buccal vs. sublingual) and, for HA-targeted systems, include CD44-status as a covariate [94,95].

Taste masking represents another formulation difficulty. Drugs that have a bitter or metallic taste may induce aversion or nausea, particularly in pediatric or geriatric populations. While taste-masking techniques such as flavoring agents, polymer encapsulation, or ion-exchange resins exist, they must not interfere with drug release kinetics or mucoadhesion [96,97].

Interindividual variability adds further complexity. Differences in saliva composition, mucosal thickness, enzymatic activity, and even the oral microbiome may affect drug absorption and therapeutic response between patients [98,99]. Conditions such as xerostomia or periodontal disease can further alter the local environment, reduce formulation effectiveness or increase side effects [99,100].

Moreover, pH fluctuations in the oral cavity, caused by diet, disease, or circadian rhythms, can affect drug solubility, ionization, and stability. For instance, weakly basic drugs may become ionized and less permeable in the relatively acidic sublingual region, necessitating the development of pH-modulating excipients or prodrugs [101,102].

The limited surface area of the oral mucosa, approximately 200 cm^2^, further constrains the volume of drug that can be administered. This is particularly problematic for drugs requiring high doses or sustained plasma levels, which may necessitate repeated administration or the development of highly concentrated formulations [7,103].

Finally, formulation retention and user experience are vital. Large or poorly designed patches may be uncomfortable or interfere with speech and swallowing. Dosage forms must be discreet, comfortable, and capable of adhering to the mucosa without causing irritation, especially for long-term or chronic therapies [104,105].

To resolve these descriptions of barriers, there is an alignment for each challenge with a GAG-centric solution path. For salivary washout and shear, wet-adhesive catechol-modified HA or chitosan and thiolated chitosan enable durable adhesion via catechol bonding and mucin disulfides, respectively; bilayer films add unidirectional flux control (design readouts: detachment force, survival under simulated flow) [106,107]. For enzymatic degradation, moderately crosslinked HA/alginate networks and GAG-coated nanoparticles provide sacrificial protection while preserving release [14]. For permeability limits, chitosan-based (including thiolated) matrices afford reversible tight-junction opening at tolerated doses, while HA–CD44 interactions support receptor-mediated uptake/retention in diseased tissues (readouts: TEER recovery, paracellular marker flux, CD44-stratified uptake) [108,109]. Finally, pH/ionic variability can be mitigated using ion-tunable alginate/HA blends and catechol-alginate surfaces that maintain adhesion in saliva [110]. 

Where chemical enhancers are considered, risk–benefit should be disclosed explicitly. For example, 10% RAMEB is cytotoxic on reconstructed buccal epithelium, whereas 2–5% appears tolerated over repeated exposures; bile salts show efficacy with concentration-dependent epithelial stress; and 0.5–2% SLS exposures are linked to mucosal desquamation in humans (design mitigation: low-dose, pulsed exposure with full TEER/LDH recovery reporting) [111]. Given real-world variability in salivary flow and mucosal status, in vitro performance should be profiled under xerostomia-mimicking and normosalivary conditions and, for HA-targeted systems, analyzed by CD44 expression [93,95].

Overcoming these limitations demands an interdisciplinary approach, combining insights from pharmaceutical sciences, biomaterials engineering, oral medicine, and patient-centered design. Advances in mucoadhesive biomaterials, nanocarrier technologies, and responsive systems (e.g., thermosensitive or pH-triggered gels) are beginning to address these challenges, offering promising avenues for the next generation of oral mucosal drug delivery platforms.

## 4. Current Dosage Forms and Technologies for Oral Mucosal Delivery

Within mucosal DDS, the focus is on retentive films/wafers that do not disintegrate during wear and, when backed, direct flux toward tissue. Dispersive formats (ODFs/lozenges) are considered separately for on-surface indications. 

### 4.1. Conventional Forms: Films, Tablets, and Sprays

Oral films, also referred to as orally disintegrating films or oral soluble films, have become highly popular due to their ease of use, particularly in pediatric, geriatric, and dysphagic populations [112]. These thin, flexible polymeric films are engineered either for rapid dissolution or adhesion to the oral mucosa, enabling both local and systemic drug delivery. Recent advancements in film technology have focused on enhancing mucoadhesion to prolong residence time and improve drug absorption. One notable innovation is the bilaminar film structure, consisting of a mucoadhesive hydrophilic polymeric layer containing the active pharmaceutical ingredient and an inert backing layer that ensures unidirectional drug release toward the buccal mucosa. Such films have been developed for rapid pain relief in clinical settings [113]. Current research also explores the development of mechanically robust films and those capable of delivering complex macromolecules such as proteins and peptides, thereby expanding their therapeutic utility [113].

While oral tablets are typically intended for swallowing, specialized oral mucosal tablets are formulated for buccal or sublingual use. These include medicated lozenges that slowly dissolve in the oral cavity and are primarily used for localized treatment, such as sore throat or local anesthesia [6]. More advanced buccal or sublingual tablets are designed to adhere to the mucosa, enabling direct systemic absorption. These formulations often employ mucoadhesive polymers to enhance contact time with the mucosal surface and improve drug uptake [114]. Key development challenges center on achieving reproducible disintegration and drug-release profiles while optimizing palatability and mouthfeel to support adherence [6,114]. Extended-release buccal tablets for indications such as labial herpes and oropharyngeal candidiasis are currently under investigation using advanced mucoadhesive technologies [112].

Oral sprays represent a convenient method for rapid drug delivery to the oral mucosa, often resulting in a swift onset of action. These systems typically consist of a liquid formulation dispensed as a fine mist onto the buccal or sublingual membranes. They are particularly useful for emergency medications requiring rapid systemic effects, such as those used for angina management [6]. Recent innovations focus on enhancing delivery precision, dose consistency, and aerosol characteristics. The ability of oral sprays to bypass first-pass metabolism and provide rapid absorption makes them a valuable option for both systemic therapies and localized treatment of oral infections and inflammatory conditions [115,116].

To aid formulation planning, Table 1 summarizes common material categories and typical ratio ranges in oromucosal MDDS. Values are literature-consistent starting points and must be adjusted by QbD/DoE for a given API, target site, and residence time.

### 4.2. Semisolid Dosage Form: Gels, Creams, and In Situ–Gelling Systems

Semisolid dosage forms represent conventional yet highly adaptable platforms for oromucosal therapy. Rheologically tuned vehicles (e.g., carbomer- or chitosan-based) can increase residence by enhancing mucosal wetting and bioadhesion, while permitting facile spreading over irregular ulcer beds and dose titration at the point of care [85]. Evidence syntheses and recent applications highlight their utility across inflammatory and infectious indications: mucoadhesive buccal gels are an active area of development, with hydrogels and nanogels explored to counteract salivary washout and improve local exposure [117]. Clinically, hyaluronic-acid (HA) gels reduce pain and accelerate healing in recurrent aphthous stomatitis, with systematic reviews and randomized trials supporting efficacy versus placebo and, in some studies, non-inferiority to topical corticosteroids [41,118]. While semisolids offer strong patient acceptability and device simplicity, their performance is limited by dilution and mechanical clearance; current research therefore prioritizes mucoadhesive polymer selection and triggerable gelation to sustain drug residence without compromising mouthfeel [117].

### 4.3. Controlled and Targeted Delivery Systems

The dynamic environment of the oral cavity—characterized by salivary flow, mastication, and continuous movement—demands advanced delivery systems capable of providing controlled and targeted drug release.

Controlled-release systems are specifically engineered to maintain therapeutic drug concentrations over extended periods, thereby reducing dosing frequency. Mucoadhesive polymers are central to these systems, as they significantly prolong residence time at the absorption site [119]. Controlled release can be achieved through innovative designs, including homogeneous drug dispersion within a polymer matrix, from which the drug is gradually released via diffusion, matrix erosion, or a combination of both [119,120]. Multilayered devices featuring distinct layers can further regulate drug kinetics and ensure unidirectional release toward the mucosa, minimizing drug loss to saliva. Examples include mucoadhesive patches with an impermeable backing layer [112,113].

“Smart” delivery systems represent a rapidly growing area of research. These platforms respond to specific physiological stimuli in the oral cavity—such as pH shifts, temperature changes, or enzymatic activity—enabling precise, on-demand drug release and improved therapeutic efficacy while minimizing side effects [84,120]. Hot-melt extrusion is an emerging manufacturing technique that enables controlled release and improved palatability without organic solvents, offering a more sustainable production method [121].

Targeted delivery systems aim to concentrate the drug at a specific site within the oral cavity or target specific cell populations, thereby enhancing local efficacy and minimizing systemic exposure and side effects. This approach is especially useful for localized treatment of oral diseases such as infections, inflammation, and cancers. Nanoparticles—including liposomes, solid lipid nanoparticles, and polymeric nanoparticles—are often used to enhance drug stability, solubility, and mucosal penetration due to their small size [122,123]. These carriers can be engineered to encapsulate a wide range of active agents, from antimicrobials to chemotherapeutics, and have shown promising results in enhancing drug retention and uptake in oral squamous cell lines [8,123].

Hydrogels and nanofibers offer versatile platforms for localized delivery: hydrogels ensure sustained release and protect encapsulated drugs, while nanofibers—owing to their high surface area and adaptability—are particularly suited for hard-to-reach areas such as periodontal pockets or mucosal wounds [122,124].

Finally, ligand-mediated targeting involves functionalizing drug carriers with ligands that bind selectively to receptors overexpressed in diseased oral tissues. This precise strategy promotes site-specific drug accumulation, reduces toxicity, and mitigates the risk of drug resistance by sparing healthy cells and focusing therapy at the desired site [125,126].

Because mucoadhesion is method-dependent and no pharmacopoeial cutoff is defined, we specify method-tied acceptance values. Using a texture-analyzer pull-off test on porcine buccal mucosa (10 N contact, 2 min; detachment 0.1 mm s^−1^), we target pull-off ≥ 0.5 N cm^−2^ and shear ≥ 2.0 N cm^−2^, consistent with reported ranges for successful buccal films (pull-off ~0.42–1.1 N cm^−2^; shear ~1.7–5.6 N cm^−2^) [127]. For residence under biorelevant hydrodynamics, we require ≥ 120 min ex vivo, noting that DOPA-modified films remained adhered for up to 8 h in flow/rotation assays [106]. Palatability should be screened with an electronic tongue and bridged to human panels, with acceptance prespecified as ≥80% “acceptable” on validated scales; e-tongue use and its limits in predicting human taste are documented [128,129]. Safety/epithelial integrity is evidenced by TEER recovery to ≥90–95% of baseline within hours in TR146/organotypic models, where TEER is a validated barrier metric [130]. Methods should reference compendial rotational viscosity/rheology procedures for reporting and we note that no mucoadhesion threshold is provided in pharmacopeias, as summarized in the Eur. Pharm. Biopharm. review article [131].

**Figure 3:**
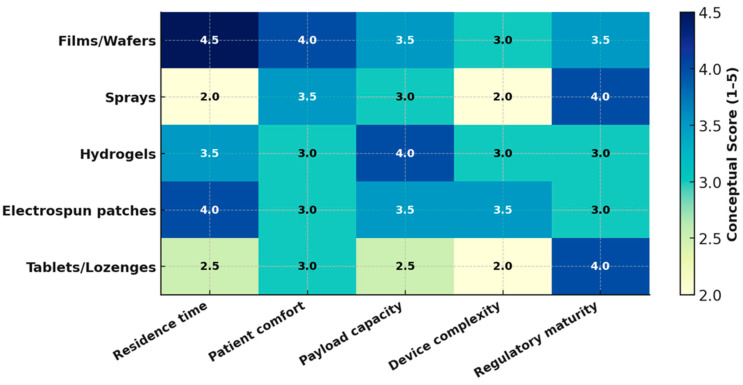
Comparative performance of oromucosal dosage forms (conceptual scores, 1–5). Scores summarize expected trends from the literature and this review; adjust to specific formulations where quantitative data are available (an original scheme based on the current literature data).

Figure 3 compares the performance of oromucosal dosage forms using conceptual scores 1–5 to summarize expected trends from the current literature.

## 5. Glycosaminoglycans in Drug Delivery

### 5.1. Hyaluronic Acid (HA)

Hyaluronic acid (HA) is a large non-sulphated glycosaminoglycan and a key component of the extracellular matrix (ECM). It is a biodegradable polymer, and due to variations in its molecular weight, HA derivatives can be formulated into creams, gels, and drops. HA has excellent potential for a wide range of applications that go far beyond facial esthetics. This wide applicability stems from its distinctive biophysical properties, including mechanical strength, swelling behavior, lubrication, tissue regeneration, and hydration. HA is found in virtually all cell types, underscoring its biological relevance. A critical aspect of HA’s activity involves its interaction with cell surface receptors, particularly the cluster of differentiation 44 (CD44) receptor, which is widely expressed in activated macrophages central to inflammatory responses. These interactions enable targeted delivery, resulting in increased cellular uptake and improved anti-inflammatory outcomes. However, a major limitation of HA-based delivery is its relatively short half-life in biological fluids. Crosslinking techniques are often employed to overcome this, though these can increase toxicity compared to “free” HA [132]. HA can be blended with other water-soluble biopolymers such as gelatin, alginate, chitosan, and cellulose derivatives to extend drug release periods from HA-based matrices [133].

### 5.2. Chitosan

Chitosan is a deacetylated derivative of chitin, a natural polysaccharide primarily found in the exoskeletons of crustaceans, insects, and fungi [134]. It is a bioactive compound exhibiting antitumor, immunoenhancing, antifungal, antimicrobial, antioxidant, and wound healing activities. Chitosan can form films, gels, nanoparticles, microparticles, and beads. It is biodegradable and metabolized into amino sugars, which are safely absorbed by the human body. These features—combined with non-toxicity, biocompatibility, biodegradability, and low cost—have led to extensive pharmaceutical applications in biomedicine and drug delivery systems [135]. Chitosan solubility depends on multiple factors, including molecular weight, degree of acetylation, pH, temperature, and polymer crystallinity [136].

### 5.3. Dextran

Dextran is a neutral, complex, branched glucan, mainly produced extracellularly from sucrose by several lactic acid bacteria [137,138]. It is widely used in nanomaterials for drug delivery due to its excellent solubility, biocompatibility, biodegradability, and non-immunogenicity [139,140]. Numerous dextran-based systems with tailored properties and geometries have been developed, including micelles, nanoparticles, nanoemulsions, magnetic nanoparticles, microparticles, and hydrogels [141]. Dextran is soluble in ethylene glycol, formamide, glycerol, methyl sulfoxide, and water. Its morphology influences water retention and gel formation. At low concentrations (2.5% *w*/*v*), it behaves like a liquid, while at higher concentrations (>5% *w*/*v*), it exhibits both gel-like and liquid-like behavior [142]. Dextran-based gels for drug conjugation have demonstrated no toxic side effects and offer high drug-loading capacity, ease of use, and stable performance. These systems can achieve prolonged circulation times and targeted drug delivery to tumors, minimizing systemic toxicity [143,144,145].

### 5.4. Alginate

Alginate is a water-soluble, naturally occurring linear polysaccharide known for its biocompatibility, biodegradability, low immunogenicity, and strong mucoadhesive properties—features that make it suitable for oral drug delivery [146]. Its viscosity, sol–gel transition, and water-uptake capacity determine its physicochemical performance [147]. Alginates are cost-effective, chemically versatile, and form hydrogels via ion-induced crosslinking with good mechanical strength [148]. Drug delivery systems based on alginate include hydrogels, microparticles, nanoparticles, and porous scaffolds. Alginate-hybrid materials (AHMs) are being developed to improve drug-loading capacity, release control, and responsiveness to environmental stimuli [149,150]. Chitosan-alginate hybrid nanoparticles have been explored for oral anti-cancer therapies. Electrostatic interactions between chitosan’s amino groups and alginate’s carboxyl groups result in stable hybrid structures capable of protecting bioactive compounds from gastric degradation [148].

### 5.5. Chondroitin Suphate

Chondroitin sulfate (CS) is a sulfated, anionic glycosaminoglycan that imparts hydration and viscoelasticity and readily forms polyelectrolyte complexes—most commonly with chitosan—yielding mucoadhesive matrices with enhanced mechanical integrity and residence on wet epithelium; CS-based oral formulations (e.g., CS–xyloglucan liquids) have demonstrated rheological synergism with mucin and barrier effects on reconstructed oral epithelia [151,152].

### 5.6. Dermatan Sulfate (DS)

Dermatan sulfate (DS), a GalNAc–iduronic-acid GAG, exhibits high-affinity interaction with heparin cofactor II and additive thrombin inhibition, underscoring both anti-inflammatory/antithrombotic potential and a theoretical bleeding liability that must be considered for intraoral use [153,154]. Translationally, DS shows preclinical benefits in wound repair and radiation-induced oral mucositis models, whereas robust intraoral clinical data remain sparse; conversely, CS already supports mucoadhesive oral devices/liquids, positioning CS as the nearer-term option for buccal formulations while DS warrants cautious, indication-specific development [155,156].

Table 2 provides a concise overview of the key characteristics of the aforementioned GAGs used in oral drug delivery. To guide platform selection, polymers were compared across mucoadhesion, permeability, stability, usability, sterilization, and clinical signal in Table 3 [23,27,34,142,152,157,158,159,160,161,162,163,164,165,166,167,168,169,170,171].

Among cationic systems, thiolated chitosan offers the strongest, wet-stable mucoadhesion with reversible tight-junction effects—but requires lysozyme-aware design and post-sterilization MW checks if γ-irradiated. HA provides excellent comfort and CD44-mediated localization in inflamed or malignant mucosa but is hyaluronidase-labile, favoring higher-MW or lightly crosslinked formats. Alginate excels as a thin, taste-neutral film/backing layer with tunable mechanics via G/M ratio and Ca^2+^ crosslinking, though clinical buccal film data remain sparse. Dextran/CS add hydration and barrier functions and form useful PECs (e.g., with chitosan), yet human oral-lesion data are limited. Dermatan sulfate/heparin possess compelling anti-inflammatory or anticoagulant bioactivity preclinically, but translation in the oral cavity is unproven and safety (bleeding) must be considered. Overall, clinical evidence currently supports HA and chitosan for symptomatic oral lesions, whereas antitumor claims for GAG carriers are preclinical and should be labeled accordingly [34,157,158,171].

## 6. Glycosaminoglycans-Based Delivery Platforms

**Figure 4:**
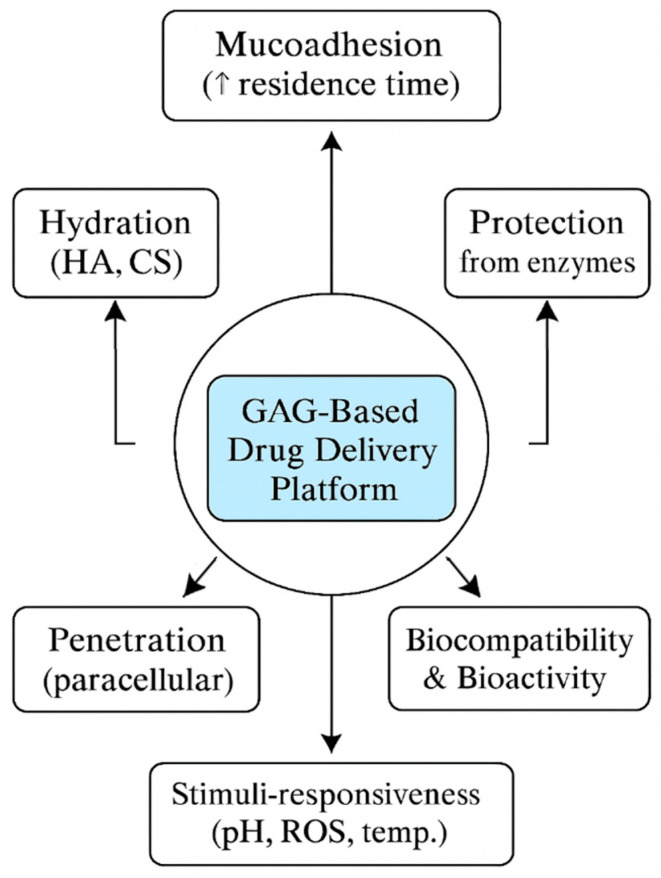
Multifunctional roles of glycosaminoglycans (GAGs) in oral mucosal drug delivery systems (an original scheme based on the current literature data). GAG-based platforms contribute to enhanced mucoadhesion, extended residence time, hydration of the mucosal surface (particularly via hyaluronic acid and chondroitin sulfate), and protection of therapeutic agents from enzymatic degradation. In addition, certain GAGs facilitate paracellular drug penetration, exhibit intrinsic biocompatibility and bioactivity, and can be engineered to respond to local stimuli such as pH shifts, reactive oxygen species (ROS), or temperature changes. Together, these multifunctional properties make GAGs highly suitable for innovative and targeted oral mucosal drug delivery strategies.

GAGs, including chitosan, HA, chondroitin sulfate (CS), and dermatan sulfate (DS), have emerged as key biomaterials in the development of drug delivery systems targeting the oral mucosa. Their unique properties—mucoadhesiveness, biocompatibility, enzymatic modifiability, and the ability to form hydrogels or composite matrices—support prolonged residence at mucosal surfaces, localized therapeutic action, and potential systemic absorption [172,173] (Figure 4). 

Over the past five years, GAG-based systems have significantly advanced across three primary platforms: mucoadhesive films (and wafers), stimuli-responsive hydrogels (and nanogels), and GAG-modified nanoparticles (and microparticles). Recently, textile-based scaffolds have emerged as a new platform type (Table 4).

### 6.1. Mucoadhesive Films and Wafers: Formulation, Stability, and Therapeutic Applications

Mucoadhesive films and wafers represent the most clinically developed format for oromucosal GAG-based drug delivery. 

Chitosan, soluble in mildly acidic aqueous media, is frequently employed; however, its pH-dependent solubility presents challenges near neutrality. To address this, salt forms such as chitosan ascorbate and chitosan glutamate are used to preserve solubility and bioadhesive properties during casting [174]. Plasticizers like glycerol are added to improve flexibility and ensure uniform drug dispersion [175]. Polyelectrolyte complexes (PECs) formed between cationic chitosan and anionic HA, pectin, or dermatan sulfate offer structural integrity without requiring chemical crosslinkers. Multilayer systems using layer-by-layer (LbL) assembly—such as alternating chitosan and HA—enable ultrathin mucoadhesive films with precise control over thickness and composition, particularly suitable for sublingual protein delivery [151,176].

Freeze-dried wafers form porous matrices that rehydrate rapidly and adhere well to mucosa. Chitosan-based lyophilized wafers have demonstrated extended residence times and high acceptability in pediatric formulations [177]. Thiolated chitosan derivatives enhance mucin binding through disulfide bond formation and improve protein stability [44].

GAG-based films are generally stable under dry conditions, although their hygroscopic nature necessitates protective packaging. One study showed that HA-based films for aphthous ulcers maintained drug content and mechanical strength over time when stabilized by freeze–thaw cycling [43]. Chitosan–ascorbate films loaded with captopril retained over 95% of drug content, FTIR and Raman spectroscopy were used to monitor changes in functional groups and network chemistry during stress studies. Degradation was assessed using stability-indicating chromatography (HPLC/UPLC with UV/RI/ELSD detection and, where required, LC–MS for identification). Polymer backbone scission was quantified by SEC/GPC (MALS/RI) to track Mn, Mw, and dispersity, with complementary NMR as needed. Residual solvents and volatile by-products were evaluated by GC(-MS) when applicable. Chitosan’s cationic nature provides high mucoadhesive strength—up to 15 N in in vitro detachment tests—sufficient to resist salivary flow and tongue movement [174]. HA, although anionic, adheres via hydration and gel-layer formation, with tunable adhesion strength achieved through freeze–thaw crosslinking cycles [43].

These delivery systems have demonstrated therapeutic utility in both local and systemic contexts. HA-based films have improved healing in aphthous stomatitis and orthodontic-related mucosal injuries [151]. Chitosan–ascorbate films for buccal captopril delivery achieved rapid systemic absorption, offering an alternative to oral tablets in hypertensive emergencies [174]. Pediatric wafers loaded with prednisolone are being developed to facilitate corticosteroid therapy in children [177]. Films delivering clotrimazole via chitosan–pectin matrices have shown antifungal efficacy in oral candidiasis, benefitting from the synergistic activity of chitosan [178].

### 6.2. Hydrogels and Nanogels: Stimuli-Responsive Matrices

Stimuli-responsive hydrogels and nanogels enable dynamic, “smart” drug release in response to environmental triggers in the oral cavity, such as pH changes, temperature fluctuations, oxidative stress, or enzymatic activity. Chemical modification of GAG backbones—exemplified by thiolation to enable disulfide exchange with mucins (enhancing interfacial bonding), catechol grafting to impart strong wet adhesion, and methacrylation to yield photo-crosslinkable networks—has underpinned the development of stimuli-responsive GAG hydrogels and nanogels [157,172]. These engineered matrices consistently show greater mucoadhesive strength and gel integrity, while enabling on-demand drug release under physiologically relevant cues—including pH/ionic strength changes, redox processes (thiol–disulfide exchange), hyaluronidase-mediated degradation (for HA-based systems), temperature, or light (photoinitiated crosslinking) [179,180,181].

Thiolated HA (HA–SH) forms disulfide bonds with mucosal cysteine residues, increasing residence time [182]. Catechol-conjugated HA, inspired by mussel adhesion chemistry, enables rapid crosslinking and strong binding to wet tissue, with successful application in oral wound dressings and antifungal films [27]. Various delivery systems have been developed:

#### 6.2.1. pH-Responsive Systems 

HA and CS, owing to their ionizable groups, support pH-dependent swelling or degradation. Zirconium-crosslinked HA hydrogels disintegrate in alkaline environments—typical of infected sites—releasing antimicrobial Zr^4+^ ions [183]. HA nanogels with pH-sensitive linkers have also been designed to protect peptides in acidic environments and release them at physiological pH [44].

#### 6.2.2. Thermo-Responsive Hydrogels

Blends of HA and Pluronic F127 form injectable gels that solidify at 37 °C, conforming to mucosal contours for localized, sustained delivery—such as for hydroxytyrosol in periodontitis models [184].

#### 6.2.3. Enzyme-Responsive Hydrogels

MMP-sensitive HA gels crosslinked with cleavable peptides selectively released doxorubicin in oral tumors, minimizing toxicity and improving targeting [173]. Bacterial protease-sensitive hydrogels have similarly been developed for periodontal therapy [185].

#### 6.2.4. Redox-Responsive Nanogels

Disulfide-crosslinked HA nanogels respond to elevated glutathione levels in inflamed or cancerous tissues, enabling intracellular drug release. These systems improved delivery of chemotherapeutics in oral cancer models [186]. Additionally, thiolated HA combined with silver–lignin nanoparticles produced a hydrogel with dual antioxidant and antimicrobial activity, useful for wound healing and mucositis [187].

#### 6.2.5. Light-Activated Systems

HA modified with photocrosslinkable groups (e.g., nitrobenzyl moieties) enables visible-light-triggered gelation within seconds, creating protective barriers over wounds [183]. In photothermal therapy, HA hydrogels loaded with indocyanine green (ICG) enabled localized heating and synergistic tumor ablation upon near-infrared light activation [188].

### 6.3. Nanoparticles and Microparticles: Targeting and Transport Strategies

GAG-functionalized nanoparticles (NPs) and microparticles (MPs) are engineered to enhance mucosal residence, facilitate epithelial transport, and enable intracellular drug delivery. HA and CS provide strong adhesion to mucins and epithelial surfaces, improving retention in the dynamic oral environment [41]. For example, HA-based nanoemulsions delivering miconazole in oral candidiasis models showed superior penetration and sustained efficacy [189]. HA-coated NPs leverage CD44 overexpression in inflamed and malignant tissues. In oral squamous cell carcinoma, HA–doxorubicin nanoparticles exhibited enhanced tumor uptake and reduced systemic toxicity [190,191]. Similarly, CS-modified carriers selectively accumulated in periodontal lesions, reducing local inflammation [192]. These carriers utilize both transcellular (via receptor-mediated endocytosis) and paracellular transport. Chitosan derivatives can transiently open tight junctions, while HA and CS maintain hydration and biocompatibility, supporting safe permeation enhancement [39]. Moreover, GAG-coated NPs have enabled buccal delivery of peptides like insulin and exenatide, achieving 12–15% relative bioavailability in diabetic rat models [193]. CS-capped gold nanoparticles also facilitated effective insulin delivery, producing plasma levels comparable to subcutaneous injection [194]. The most commonly used colloidal nanoparticles in medical materials include colloidal silver particles (10 nm) and zinc oxide nanoparticles (100 nm) [195]. Although still largely in preclinical stages, HA-based mucoadhesive films and nanoparticles have undergone clinical testing for ulcer healing and mucositis relief, confirming their safety and efficacy [44]. Emerging hybrid systems—combining GAG-based nanoparticles with hydrogels or wafers—are being developed to optimize both retention and tissue penetration.

Across films, hydrogels, and particulate systems, GAG-based platforms for oral mucosal delivery offer multifunctional capabilities, including strong adhesion, structural flexibility, and responsiveness to pathological microenvironments. These natural polymers not only enhance pharmacokinetics but also contribute therapeutic benefits through inherent bioactivity. As these technologies progress toward clinical use, their integration into personalized, non-invasive therapies for oral and systemic diseases becomes increasingly feasible.

In vivo success for oral mucosal systems hinges less on exotic chemistries and more on mastering mechanics + hydrodynamics + manufacturability. Light-activated formats and “injection-like” systemic claims remain promising but are not yet substantiated in the oral cavity; near-term translation will come from devices that document real-world stability, comfort, saliva-robust residence, and GMP-credible scale-up [196].

### 6.4. In Vivo Challenges, Evidence Gaps, and Scale-Up Considerations

Devices designed for oral mucosa application must withstand intermittent compressive loads (hundreds of newtons during mastication) and continuous shear from the tongue and cheeks without edge-lift or fracture. Practically, buccal films should be profiled for tensile strength, puncture/tear resistance, peel strength on wet substrates, and cyclic bending in artificial saliva; these metrics predict handling and wear behavior in vivo. All studies should report peak peel force (N), energy to detach (N·mm), and survival (min) under simulated salivary flow with controlled shear [197,198]. Along with mechanical properties to overcome mucosal flexibility, thickness, surface roughness, and taste/aftertaste govern acceptability; “acceptable” in children and older adults correlates with thin, smooth, non-friable films that place and stay discreetly. Include human-factor style endpoints: successful first-time placement (%), speech interference scores, perceived mouthfeel, and time to forget device is present. Map these to formulation knobs (polymer ratio, plasticizer, surface finish). Early clinical and panel studies on orodispersible/buccal films provide workable acceptability frameworks you can adapt [82,199]. In situ light-triggered gels and adhesives are attractive for on-demand setting and spatial control, but most data derive from dental resin processing or non-oral tissues; oral drug-delivery trials are scarce. If light activation is pursued, align photoinitiator choice and dose with dental safety practice (e.g., blue-light–activated camphorquinone systems), characterize heat rise, and verify cytocompatibility on oral epithelium models. It is important to address and acknowledge that clinical evidence remains extrapolative for medications (vs. restorative resins), and position claims accordingly [196]. There are three recurring bottlenecks limit translation that need to emphasize: (i) dose and content uniformity across large sheets, (ii) residual solvents and extractables, and (iii) sterilization that preserves polymer MW and mechanics. [83,200].

## 7. Analytical Methods in the Evaluation of Oral DDS

### 7.1. Structural Characterization of GAGs

The structural complexity of GAGs makes them particularly difficult to analyze. Mass spectrometry (MS) has become a key tool in this field due to its exceptional sensitivity, ability to detect subtle structural variations, and capacity to handle complex biological mixtures. Therefore, many studies show that researchers are gaining insight into GAG structures and connecting them to their biological roles, especially regarding their interactions with proteins, as past studies have highlighted the importance of GAG structure-function relationships [201].

GAGs exhibit significant molecular heterogeneity, particularly in their uronic acid, hexosamine components, and sulfate group positions. Understanding their biological roles requires detailed detection and structural identification. Extraction typically involves enzymatic depolymerization using exogenous proteinases or sodium hydroxide. Physicochemical analyses are performed after enzymatic treatment, using techniques like ion-pair chromatography and MS [202].

The structural characterization of GAGs is complex, requiring enzymatic depolymerization with specific bacterial enzymes followed by disaccharide analysis using Gel Permeation Chromatography (GPC) [203], High-Performance Liquid Chromatography (HPLC) [204], or Ultra-Performance Liquid Chromatography (UPLC) [205], Nuclear Magnetic Resonance (NMR) spectroscopy [206], Capillary Electrophoresis (CE) [207], and Fluorophore-Assisted Carbohydrate Electrophoresis (FACE) [208].

Liquid chromatography-mass spectrometry (LC-MS) and MS have become prevalent due to their ability to analyze GAGs without interference from biological impurities. Reverse-phase ion-pair Reverse-Phase Ion-Pair High-Performance Liquid Chromatography (RPIP-HPLC) employs volatile ion-pairing reagents that allow analytes to bind to hydrophobic stationary phases and remain compatible with electrospray ionization (ESI)-MS [209]. This approach suits a range of GAGs, from unsulfated to highly sulfated. Reverse-Phase Ion-Pair Ultra-Performance Liquid Chromatography–Mass Spectrometry (RPIP-UPLC-MS) further improves resolution, sensitivity, and efficiency using high-pressure columns with small particle sizes. However, factors such as ion-pair reagent concentration, counter-ion type, and pH can influence separation outcomes [210]. Routine GAG structural analysis often requires multiple enzymatic treatments, disaccharide isolations, chromatographic steps, and various MS detection methods, making it labor-intensive.

MS techniques like ESI and matrix-assisted laser desorption/ionization (MALDI) are widely used for structural analysis of GAG oligosaccharides. MALDI time-of-flight (TOF) MS and Electrospray Ionization Mass Spectrometry (ESI-MS) effectively analyze large polar macromolecules [211]. NMR spectroscopy also offers detailed structural insights, including saccharide composition and sulfation patterns, but requires relatively large amounts of purified GAGs, limiting its routine use.

Accurate analysis of GAG structures continues to pose major analytical challenges [212]. Unlike proteins and nucleic acids, which can be amplified or overexpressed, GAGs are synthesized through a non-template enzymatic process. This begins with a uniform copolymer that is later heavily modified by enzymes such as deacetylases, sulfotransferases, and epimerases, resulting in heterogeneous chains with varying levels of acetylation and sulfation. These modifications produce highly complex and diverse biological GAG mixtures, typically available only in limited quantities. Because of their high molecular weight and low abundance, analytical methods like NMR or X-ray diffraction are often impractical. This is why developing advanced MS techniques for GAG analysis has attracted significant research attention. Two key features of GAGs—their negative charge and the delicate nature of their sulfate groups—strongly influence the choice of MS methods.

Over the past decade, improvements in online separation techniques, ion activation methods, and software for automated MS/MS data interpretation have greatly advanced GAG structural analysis [213]. Given their involvement in essential biological functions such as cell signaling, wound repair, and blood coagulation, GAGs are crucial targets for detailed structural studies.

Most linear GAG chains are built from repeating disaccharide units made up of a hexosamine and a hexuronic acid, with keratan sulfate (KS) being the main exception, as it contains hexosamine and galactose instead. Even without their attached protein cores, GAGs are highly intricate and varied molecules due to the wide range of possible combinations in chain lengths, sugar sequences, chemical compositions, sulfo group placements, and how these domains are arranged along the chain. The specific types of GAG chains—such as heparin/heparan sulfate (Hp/HS), chondroitin/dermatan sulfate (CS/DS), and keratan sulfate (KS)—are defined by their characteristic repeating disaccharide units, and these structural features play a key role in shaping how proteoglycans (PGs) are organized [214]. New MS methodologies, including innovations in sample preparation and tandem MS approaches, have led to impressive gains in the accuracy and speed of GAG characterization.

### 7.2. Drug Release Studies and Permeation Assays

The development of oral drug delivery systems using biopolymers such as GAGs—including hyaluronic acid, chitosan, dextran, and alginate—has gained significant traction due to their biocompatibility, biodegradability, and mucoadhesive properties. However, evaluating these materials requires a multi-layered analytical approach that rigorously examines their performance, stability, and safety.

Synthetic membranes are often preferred over biological tissues because they are more readily available, cost-effective, and structurally simpler, allowing for large-scale studies and mechanistic investigations. Additionally, they provide more reproducible permeation data by eliminating in vivo variables such as skin age, sex, ethnicity, and anatomical location. Despite these advantages, artificial membrane studies still show notable variability [215].

These assays help determine the drug release profile—whether immediate, sustained, or controlled—under simulated gastrointestinal conditions. Permeation assays across intestinal models (such as Caco-2 cell monolayers or ex vivo tissues) provide insight into the ability of the polymer–drug system to cross epithelial barriers, a crucial step for oral bioavailability.

To reflect GAG chemistry, dissolution/release tests were adapted as follows: (i) mucin-containing saliva simulants to capture rheological synergism that predicts mucoadhesion and residence; we report ΔG′/viscosity shift alongside release (bioadhesive liquids show positive synergy with mucin) [154]; (ii) Calcium dynamics for alginate systems: internal gelation using CaCO_3_/GDL and phosphate/ionic-strength challenges to map crosslink stability and release (alginate release is sensitive to Ca^2+^ availability and competing anions) [216,217,218]; (iii) Enzyme-triggered conditions (e.g., hyaluronidase for HA networks) to evaluate on-demand degradation-mediated release [219]. 

Permeability refers to how easily a molecule can pass through a biological membrane. It is typically measured as a velocity, expressed in units such as centimeters per second (cm/s), representing the distance the molecule travels across the membrane per unit time. This measurement applies regardless of whether the molecule moves via active transport or passive diffusion mechanisms [220].

For transport and safety, TR146 oral epithelium is a relevant buccal model (with TEER and paracellular markers), while Caco-2 can be used as a comparative tight-junction reference [130]. Chitosan and thiolated chitosan can transiently open tight junctions and increase permeation under controlled conditions, so TEER recovery should be documented [221]. For hyaluronan carriers, CD44-aware uptake or retention assays are justified because CD44 is present and can be upregulated in oral tissues [222,223]. Cell-free artificial barriers (for example, PermeaPad) are best used as screening tools and cross-checked against TR146 or ex vivo buccal tissue [224]. Mucin interaction and ionic crosslinking govern adhesion and release for many anionic GAG matrices (for example, alginate), and thiolated chitosan adds reversible tight-junction modulation; ignoring these features can misestimate real-world performance [152].

Critically, many studies focus on release kinetics (zero-order, first-order, Higuchi, or Korsmeyer–Peppas models) but often lack in-depth correlation with in vivo pharmacokinetics, highlighting a gap between bench testing and clinical translation.

### 7.3. Bioadhesion Testing and Mucoadhesive Properties

The need to better understand the functions and mechanisms of action of GAGs has driven the development of both qualitative and quantitative analytical techniques. These include classical staining methods like alcian blue and toluidine blue, as well as separation techniques such as paper chromatography, thin-layer chromatography, gas chromatography, HPLC, and capillary electrophoresis. In addition, advanced methods such as the 1,9-dimethylmethylene blue assay, enzyme-linked immunosorbent assays (ELISA), and mass spectrometry have been developed to provide more precise and detailed analyses of GAGs [225].

Despite promising in vitro results of bioadhesion tests, a major challenge lies in standardizing these tests, as variability in biological tissues and testing conditions can result in poor reproducibility and limit direct comparisons across studies [226]. Mucoadhesion is commonly described as a two-stage process. In the contact stage, the formulation wets and makes intimate contact with the mucus layer; viscosity, surface energy, and initial polymer–mucin interactions (electrostatics, hydrogen bonding) dominate. The consolidation stage follows, in which polymer chains interpenetrate the mucus network and stronger interactions form (secondary bonding, ionic bridges, or covalent linkages), increasing the work of adhesion and resistance to shear [227]. Relative to neutral matrices (e.g., HPMC/PVA), GAGs offer additional adhesion mechanisms. (i) Electrostatics and hydration: anionic HA/alginate and cationic chitosan provide high interfacial hydration and charge-mediated attraction to mucins; polymer–mucin rheological synergism (ΔG′ or viscosity gain) is frequently observed and correlates with longer residence [228]; (ii) Covalent/coordination bonding: thiolated chitosans form disulfide bridges with mucin cysteines, and catechol-grafted GAGs enable wet adhesion via catechol oxidation/coordination, both increasing adhesive strength beyond hydrogen-bonding alone [157]; (iii) Ionic crosslinking/bridging: alginate networks stabilized by Ca^2+^ (including CaCO_3_/GDL systems) resist dilution and can maintain contact under flow, a consolidation effect sensitive to calcium/phosphate balance. (iv) Bio-specific retention: HA–CD44 interactions add a receptor-mediated component to consolidation in inflamed or diseased oral tissues where CD44 is expressed or upregulated [222]. Applied examples reflect these mechanisms: thiolated chitosans consistently show higher detachment forces than unmodified chitosan; catechol-modified chitosan/HA systems improve wet adhesion on oral models; and chondroitin-sulfate–based oral liquids exhibit polymer–mucin synergism and barrier effects on reconstructed epithelia [229].

Both soft (mucosal) and hard (enamel and dentin) tissues in the oral cavity are rapidly coated by the acquired oral pellicle within minutes, beginning with an electron-dense protein layer followed by a more complex globular layer [230].

This pellicle consists mainly of selectively adsorbed salivary proteins and peptides, but it also includes components from gingival crevicular fluid, blood, bacteria, mucosal cells, and dietary substances. It plays multiple protective roles: acting as a lubricant, shielding the dental surface, preventing decalcification, and providing antibacterial defense through enzymes such as lysozyme and peroxidases. However, it also contains molecules that facilitate bacterial adhesion—such as glycolipids, fibrinogen, and collagen—which support the attachment of early colonizers like *Streptococcus* and *Actinomyces*, initiating biofilm formation. Bacterial adhesins mediate this binding, and the final biofilm composition varies by location in the mouth (e.g., supragingival vs. subgingival) and between individuals. Nevertheless, studies indicate no major differences in pellicle structure or protein profile between caries-active and caries-inactive subjects [231].

### 7.4. Stability, Physicochemical Characterization, and Biocompatibility

Stability assessment—including storage stability and drug–polymer compatibility—is essential for ensuring the practical viability of oral formulations. Techniques such as dynamic light scattering (DLS) provide critical data on particle size distribution and zeta potential, which are directly linked to colloidal stability, aggregation behavior, and mucoadhesive performance. Although many studies report favorable initial physicochemical profiles, long-term stability data under physiological and storage conditions are often underreported, potentially limiting scalability and clinical translation.

Physicochemical characterization of GAG-based MDDS should link material identity to performance. Functional groups and crosslink signatures are verified by FTIR/Raman, while molecular-weight distributions (and degradation fragments) are best quantified by SEC coupled to multi-angle light scattering (SEC-MALS) [232,233]. Viscoelastic behavior and viscosity are measured with rotational rheometry following pharmacopeial guidance (USP <912>) and, for mucoadhesive systems, complemented by mucin–polymer “rheological synergism” (ΔG′/viscosity gains) as a mechanistic proxy for adhesion. For nanoscale carriers (nanogels), dynamic light scattering provides hydrodynamic size and swelling behavior in relevant media [234,235]. Finally, chemistry-based safety tests include residual solvent quantification per ICH Q3C (R9), aligned to permitted daily exposures and validated analytical methods [236,237].

Capillary electrophoresis (CE) is highly sensitive and efficient, offering rapid separation and compatibility with multiple detection methods, such as UV spectroscopy, MS, NMR, and Laser-Induced Fluorescence Detection (LIF) [238]. While CE-LIF is highly effective, it has traditionally required multiple enzymatic digestion and separation steps, complicating the workflow. Recent advancements in CE-LIF protocols have simplified the process, enabling higher throughput by allowing analysis of GAG-derived disaccharides in a single run [239].

FACE, which uses derivatization with fluorophores such as 2-Aminoacridone (AMAC), offers high sensitivity and allows simultaneous analysis of multiple samples [240]. FACE can detect disaccharides from extremely small sample volumes and has been validated as highly selective and accurate for chondroitin sulfate (CS) in biological matrices. It also enables concurrent separation of CS/dermatan sulfate (DS) and hyaluronan, making it a powerful tool for analyzing low-abundance sulfated disaccharides.

For GAG-based mucosal drug-delivery systems, biocompatibility should follow a risk-based ISO 10993-1 strategy for surface-contacting devices, selecting endpoints by contact type/duration (e.g., cytotoxicity, irritation, sensitization) [241]. Cytotoxicity is typically shown by ISO 10993-5 [241] and USP <87> in vitro reactivity assays; irritation/sensitization are addressed per the ISO 10993 framework [241]. Because the target tissue is oral epithelium, reconstructed or cell-line models (TR146) are appropriate to confirm epithelial integrity (e.g., TEER recovery) and buccal tolerance [130]. Chemistry-based safety complements biology: residual solvents are controlled per ICH Q3C (R8/R9) and elemental impurities per ICH Q3D (R2), with testing aligned to permitted daily exposures [237,242]. FDA’s ISO 10993 guidance and endpoint matrices can be used to justify the overall test plan and document acceptability for intraoral contact [241].

By combining different analytical techniques—such as enzymatic depolymerization, chromatography, MS, CE, and FACE—researchers can achieve detailed qualitative and quantitative assessments of GAG structure and behavior, despite their inherent complexity and heterogeneity.

These techniques are frequently employed to characterize chemical structure, confirm drug loading, and detect degradation products. When combined with biological assays—such as cytotoxicity tests (methyl thiazolyl tetrazolium (MTT), lactate dehydrogenase (LDH)), hemolysis assays, and histocompatibility studies—they form a comprehensive toolkit for validating the safety and performance of GAG-based materials.

Nonetheless, there is a critical need for more advanced and standardized in vivo biocompatibility testing, particularly due to the complex degradation pathways that natural polymers may undergo in the gastrointestinal tract. While analytical methods for evaluating GAG-based oral drug delivery systems have significantly advanced, key challenges remain in harmonizing in vitro and in vivo results, standardizing bioadhesion and permeation assays, and expanding stability studies under realistic storage and usage conditions. A more integrated application of cutting-edge techniques—such as high-resolution MS, atomic force microscopy (AFM), and in situ imaging—could further deepen mechanistic understanding and accelerate the clinical translation of these promising biomaterials.

### 7.5. Microscale Imaging and Characterization Methods

Several analytical techniques have been employed to elucidate the bonding mechanisms between dental hard tissues, luting agents, and restorative materials. Among these, AFM, though widely used in materials science, remains underutilized in dental research despite its extensive capabilities. AFM offers atomic-level resolution with minimal sample preparation, making it well-suited for investigating dental substrates. It has been widely applied in studies characterizing enamel and dentin erosion. More recently, AFM nanoindentation has enabled detailed assessments of the mechanical properties and demineralization processes of enamel [243].

Although AFM is most commonly used to obtain topographic images of surfaces, it includes dozens of modes—both basic and advanced—that can reveal additional properties of biomaterials. In dental research, these capabilities are rarely explored. Techniques such as phase-contrast imaging, force-distance curve analysis, nanomechanical mapping, and Kelvin Probe Force Microscopy (KPFM) allow for the exploration of topological, mechanical, and electrical properties of modified Y-TZP (yttria-stabilized tetragonal zirconia polycrystal) surfaces. These advanced AFM modes provide a deeper understanding of surface interactions, enabling accurate characterization of adhesive features [244]. As such, AFM emerges as a vital interdisciplinary tool, bridging solid-state physics, microbiology, and dental materials science [245].

In addition to AFM, other high-resolution imaging methods such as Scanning Electron Microscopy (SEM), Energy-Dispersive X-ray Spectroscopy (SEM-EDX), and Transmission Electron Microscopy (TEM) are widely used. SEM provides detailed visualization of the surface morphology of dental hard tissues and is particularly effective for analyzing enamel. Proper sample preparation—including drying, embedding, sectioning orientation, and acid etching—is essential to ensure optimal imaging results. These parameters are especially important when observing small specimens or multiple planes within a single sample [246].

SEM-EDX, a combined imaging and elemental analysis technique, is commonly employed to examine enamel surface morphology and quantitatively assess the calcium-to-phosphorus (Ca/P) ratio—an important marker of enamel integrity. The crystalline structure and Ca/P ratio are essential for evaluating the effects of remineralizing agents. A recent study by Raj demonstrated that enamel demineralization in the control group (exposed to an acidic pH < 5.5) led to a decrease in the Ca/P ratio. Four remineralizing agents were tested in comparison, and an increased Ca/P ratio was associated with more effective enamel recovery. Group II demonstrated the highest mineralization efficacy after 21 days, followed by Groups I, III, and IV. SEM imaging at 1500× magnification revealed partial crystal recovery and re-establishment of the interprismatic enamel structure in Groups I and II, with Group II showing superior surface uniformity and smoothness [247].

To reduce methodology reporting, each characterization was selected for its predictive value against an in-mouth barrier: oscillatory rheology (G′/G″, LVER, ΔG′ with mucin) predicts residence time and washout resistance; texture analysis (detachment work/force) quantifies adhesion under shear; and TR146/OME assays (TEER recovery, permeability, viability) balance permeation gains with epithelial safety. All rheology methods should follow compendial guidance (USP <911>/<912>, Ph. Eur. 2.2.8) and report geometry, temperature, and conditioning to minimize lab-to-lab variability [25]. Where possible, rheological synergism (ΔG′ with mucin) and TA detachment should co-trend with in vitro residence under flow and clinical proxy outcomes (e.g., barrier/soothing effects in HA/CS liquids), providing a transparent chain from method to barrier mitigation to patient-relevant performance [152].

Table 5 presents rheology studies of GAG systems for oral mucosa, reporting details and barrier relevance to reduce lab-to-lab variability [152,248,249,250,251,252].

## 8. GAG-Based Fibrous and Textile Platforms for Oromucosal Delivery

Textile-inspired biomaterial platforms, including electrospun nanofiber mats and woven fiber scaffolds, are emerging as innovative solutions for oral mucosal drug delivery and tissue engineering. These fiber-based scaffolds mimic key features of the native ECM while offering tunable drug loading and mechanical properties. Recent studies highlight their efficacy, biocompatibility, and translational potential in regenerating oral mucosa and delivering therapeutics. In these systems the functional matrix or finish is a glycosaminoglycan (GAG), most commonly chitosan (including thiolated forms), hyaluronan (native or derivatized), alginate, chondroitin sulfate, or heparin/HS, used alone or with an electrospinnable carrier (e.g., PCL, PLGA, PVA/PEO, gelatin) to ensure fiber formation and handling [45]. Below, we review current findings and advancements in four areas: (1) electrospun nanofibers and woven scaffolds, (2) multi-drug loading potential, (3) structural and mechanical advantages, and (4) integration with mucopolysaccharides.

### 8.1. Electrospun Nanofibers and Woven Scaffolds

Electrospinning yields sub-micron, highly porous mats that mimic the fibrous architecture of oral mucosa, maximizing surface area for intimate tissue contact and efficient drug release [253,254]. These conformable meshes adhere to wet tissue and help overcome short residence times and oral mechanics; mucoadhesive electrospun patches (e.g., PVP/Eudragit RS with dextran/PEO) show strong adhesion on porcine buccal mucosa and enable site-directed, largely unidirectional delivery [255,256]. Clinical translation is under way (e.g., AFYX mucoadhesive fiber patches) [257]. In parallel, woven/knitted/braided textiles provide durable matrices with controlled porosity; sustainable, biodegradable coatings for cellulosic fibers are being explored for oral applications [258]. Clinically, PGA fiber sheets secured with fibrin glue act as temporary matrices that reduce pain, bleeding, and contraction after oral surgery (MCFP technique) [259]. Hybrid constructs combining an electrospun layer with a textile backing leverage nano-scale tissue contact and macro-scale strength; multilayer electrospun–braided scaffolds have shown favorable early clinical performance [260,261]. Chitosan–PEO nanofibers show pronounced swelling-assisted mucoadhesion on oral models and can be engineered as bilayers with hydrophobic backings to bias flux toward the mucosa. Drug-loaded patches (for example, lidocaine) achieve rapid onset with sustained local exposure and have been characterized by spatial mass-spectrometry imaging and ex vivo buccal permeation. Hyaluronan and alginate can be electrospun via blending or derivatization and then stabilized by photo- or ionic crosslinking; alginate fibers typically require Ca^2+^ crosslinking and remain sensitive to calcium/phosphate balance [46,262]. Textile-inspired patches use meshes or fabrics finished with GAG layers (e.g., chitosan, hyaluronan, alginate) via carbodiimide coupling, ionic gelation, or layer-by-layer deposition to confer wet adhesion, hydration, and bioactive binding on mechanically robust substrates. Recent biomaterials studies demonstrate antimicrobial or anti-inflammatory payload delivery with acceptable cytocompatibility on oral cell models, supporting feasibility for local therapy [45].

### 8.2. Multi-Drug Loading Potential

Textile-inspired platforms readily accommodate combination therapy via blending, layering, or coaxial (core–shell) electrospinning, enabling programmable, multi-agent release for multifactorial oral lesions [263,264]. A representative example is a bilayer electrospun patch for dry socket prevention that co-delivered bupivacaine and prednisolone from PVP/Eudragit and PNIPAM fibers with a protective PCL backing, achieving 24 h sustained release and effective permeation and bioactivity in a tissue-engineered gingiva model [265,266]. Related designs combining antimicrobials with pro-healing cues illustrate the flexibility of fiber platforms for tailored multi-modal therapy [267]. The fibrous patch provided a sustained release of both drugs over 24 h and adhered well to the socket site, preventing loss of the blood clot. In a tissue-engineered human gingiva model, the released bupivacaine and prednisolone permeated the epithelium effectively and retained their bioactivity, producing analgesic and anti-inflammatory responses in vitro. These dual-drug patches thus simultaneously address pain and inflammation, two major issues in oral wound healing. Notably, the study concluded that such dual electrospun patches show clear potential as mucoadhesive coverings that deliver multi-modal therapy to oral wounds [266]. The success of this strategy highlights how textile-based systems can be engineered for combination therapy, improving therapeutic outcomes. Other research has combined bioactive molecules in fibers to enhance healing; for example, an electrospun scaffold carrying both an antibiotic and a growth factor could treat oral infections while promoting tissue repair (concept supported by recent designs of multi-layer nanofibers) [267]. As these examples illustrate, multi-drug loading in fiber platforms offers a powerful means to tackle the multifactorial nature of oral mucosal diseases, with the flexibility to tailor release profiles for each agent.

### 8.3. Structural and Mechanical Advantages

Oral devices must tolerate saliva, tongue shear, and intermittent loading while maintaining tissue contact. Interlaced textiles (woven, knitted, braided) provide high tensile/tear strength and dimensional stability, and their compliance can be tuned toward soft-tissue profiles relevant to oral mucosa [261,268]. Pairing a nanofiber mat with a stronger textile layer improves durability and selective permeability for exchange while maintaining a protective barrier [269]. Scaffold geometry is designable: textiles offer defined pore sizes to aid cell ingress, while electrospun mats contribute nano-topography; emerging approaches add micro-porosity to electrospun layers for deeper infiltration without sacrificing surface cues [270,271]. Clinically, PGA sheets have remained intact throughout mucosal healing, supporting stable coverage until re-epithelialization [270]. Overall, fiber-based platforms integrate biomimetic architecture with practical strength for oral repair.

Most GAG-based fibrous/textile platforms remain preclinical: clinical claims are limited to feasibility and acceptability, with controlled human pharmacodynamic or comparative-effectiveness data still scarce. Priorities include standardized mucoadhesion/permeation methods under salivary hydrodynamics, enzyme/ion-aware release protocols for HA/alginate, and scale-up strategies for solvent handling, fiber stabilization, and laminate/backing integration under GMP [45,48].

### 8.4. Integration with Glycosaminoglycans

Incorporating glycosaminoglycans into textile scaffolds has emerged as an effective strategy to improve biocompatibility, bioactivity, and mucoadhesion for oral applications [272]. By coating or blending scaffolds with these molecules, adhesion to the wet mucosal surface is enhanced, and the residence time of a drug delivery device is prolonged. In the electrospun patch developed by Santocildes-Romero et al. [266], adding dextran to the fiber formulation significantly improved the patch’s mucoadhesion in vivo.

Chitosan can be integrated with electrospun fibers either as a blended component or as a post-spinning coating. Choi et al. [273] developed a composite oral patch by dip-coating an electrospun drug-loaded mat with a chitosan layer. The cationic chitosan not only adhered well to the negatively charged mucosal surface but also served as a reservoir for the drug (in this case, human growth hormone, hGH). As hGH was released from the fibers, it became bound to the chitosan layer via ionic interactions, creating a sustained delivery system. In a canine oral ulcer model, this chitosan-coated nanofiber scaffold greatly accelerated mucosal healing: ulcers treated with the hGH-loaded chitosan-fiber patch showed significantly faster epithelial regeneration compared to patches without hGH.

This study highlights how coupling a fiber scaffold with a mucopolysaccharide component can synergistically improve therapeutic efficacy—the chitosan provided a mucoadhesive and biocompatible interface, while the loaded growth factor actively stimulated tissue repair, as supported by several other studies [48]. Electrospun nanofibers surface-functionalized with HA have also been explored to improve cell seeding and proliferation on the fibers. By grafting or adsorbing HA onto nanofibers, researchers created a more cell-friendly, hydrated interface that encourages epithelial cells to attach and spread [47,274].

Chondroitin sulfate in the previously mentioned collagen scaffold has been noted to reduce fibrosis and inflammation in regenerative templates. Likewise, chitosan possesses intrinsic antibacterial and hemostatic properties that are beneficial for oral wound healing [275]. In summary, integration of mucopolysaccharides with textile-inspired scaffolds yields composite biomaterials that combine the structural strength of fibers with the biological functionality of glycosaminoglycans. These composites better recapitulate the native oral mucosal environment, leading to improved cell adhesion, controlled inflammation, and faster tissue regeneration [272,276]. Ongoing innovations in this area include electrospinning of hybrid fibers incorporating GAG derivatives and layer-by-layer assembly of polysaccharide coatings on fiber meshes. Such strategies are pushing the field toward scaffolds that are not only mechanically suitable and drug-loadable, but also inherently instructive to cells—a key step for translating oral mucosal tissue engineering into clinical success.

In short, the present body of evidence is preclinical-heavy; the most ambitious functions—multi-drug sequencing, triggerable release, and tumor-targeted delivery—are supported by in vitro/tissue-engineered and animal studies, and will require controlled human trials with patient-centric endpoints before clinical claims can be sustained.

## 9. Clinical Applications and Future Therapeutic Directions

### 9.1. Local Therapy: Candidiasis, Ulcers, Mucositis, and Oral Cancer

GAG-based systems have shown considerable promise in the local treatment of oral mucosal pathologies such as candidiasis, aphthous ulcers, chemotherapy-induced mucositis, and oral cancer. These applications leverage the mucoadhesive, anti-inflammatory, and tissue-healing properties of natural GAGs—particularly HA and chitosan—to prolong drug contact with the affected area and support tissue regeneration while minimizing systemic side effects [44,277].

In oral candidiasis, HA-enhanced nanoemulsions and chitosan–pectin polyelectrolyte films have been utilized to deliver antifungal agents such as miconazole and clotrimazole [178,190]. These GAG-containing formulations not only increase drug retention on the buccal mucosa but also improve drug permeation into the fungal biofilm, thereby enhancing antifungal efficacy [178]. In vitro studies and early clinical observations suggest that chitosan itself may exhibit synergistic antifungal activity, attributed to its membrane-disrupting properties and its ability to reduce Candida adhesion [44].

For aphthous ulcers and oral mucositis, HA-based mucoadhesive films and gels have demonstrated significant clinical benefit. A randomized trial comparing an HA/polyvinylpyrrolidone gel with placebo found accelerated healing and reduced pain in patients with orthodontic appliance-related ulcers [44]. In chemotherapy-induced mucositis, high-molecular-weight HA has been shown to reduce epithelial apoptosis and inflammation both in vitro and in vivo, likely due to its antioxidant and cytoprotective effects on keratinocytes [108]. Pediatric trials using HA sprays (e.g., Mucosamin^®^) have also reported shortened mucositis duration and improved tolerability [278].

The therapeutic role of GAG-based systems is expanding into the oncologic setting. In a 2024 preclinical study, an electrospun HA nanofiber membrane co-loaded with methotrexate and glycyrrhizin exhibited high mucoadhesion and sustained chemotherapeutic release at the tumor site [279]. This dual-action platform promoted apoptosis of oral squamous carcinoma cells while simultaneously mitigating chemotherapy-associated mucosal damage via glycyrrhizin’s anti-inflammatory effects [280]. The targeting capability of HA, via CD44 receptor interaction, further enhanced localization of drug release to malignant tissues [281]. Such dual-functional systems highlight the translational value of GAGs in precision oncology, particularly for head and neck malignancies where localized therapy is highly desirable.

Overall, these findings demonstrate that GAG-based delivery platforms offer a non-invasive, biocompatible, and therapeutically synergistic approach for managing a wide spectrum of localized oral pathologies. As their clinical validation progresses, these systems are expected to supplement or replace traditional mouthwashes, gels, and lozenges, especially in chronic or recurrent cases.

### 9.2. Systemic Delivery: Hormones, Peptides, and Insulin

While the oral mucosa has traditionally been targeted for local therapy, it is increasingly being explored as a route for systemic drug delivery—particularly for biologics and small-molecule drugs with poor gastrointestinal stability or extensive first-pass metabolism [76]. GAG-based delivery systems, especially those incorporating HA and CS, have demonstrated promising capabilities in overcoming key mucosal barriers and facilitating transmucosal absorption of hormones and peptides [44].

Among the most studied examples is the buccal delivery of insulin. HA-modified nanoparticles have been used to encapsulate insulin in protective matrices that resist enzymatic degradation in the oral cavity. These nanoparticles adhere to the mucosal surface via mucoadhesive interactions and release insulin in a controlled manner, allowing for absorption through both paracellular and CD44-mediated transcellular pathways. In diabetic rat models, such systems achieved significant reductions in blood glucose levels and improved plasma insulin concentrations, with reported bioavailability reaching 12–15%—a marked improvement over unprotected oral insulin [282].

Similar strategies have been applied to other peptide drugs, such as exenatide (a GLP-1 receptor agonist), calcitonin, and desmopressin. GAG-based nanocarriers facilitate their stabilization in the oral cavity and prolong contact time at the absorption site, allowing for effective transmucosal uptake. Co-formulation with permeation enhancers, such as bile salts or surfactants, has been shown to further increase transport efficiency without compromising mucosal integrity [283].

In addition to peptides, hormone therapies—including estradiol and testosterone—have been formulated using GAG-based films and gels for buccal or sublingual administration. These systems offer discrete, non-invasive delivery options with steady plasma profiles, improved patient compliance, and reduced hepatic metabolism. HA and CS have been particularly favored in these applications due to their ability to form stable mucoadhesive matrices that conform to the mucosal surface and control drug release kinetics [44].

Preclinical models and early-phase clinical trials suggest that GAG-functionalized nanocarriers may also hold potential for transmucosal vaccine and immunotherapy delivery. HA-coated nanoparticles carrying peptide antigens or nucleic acids have demonstrated mucosal uptake and immune activation in animal models, providing a needle-free platform for immunization against oral or systemic pathogens [284].

Collectively, these developments highlight the growing potential of GAG-based platforms for systemic delivery via the oral mucosa. By enabling non-invasive administration of labile or poorly absorbed therapeutics, these systems may significantly expand the therapeutic repertoire of oral transmucosal delivery in endocrine, metabolic, and infectious disease management.

### 9.3. Personalization and Smart-Release Platforms

The integration of responsive and customizable technologies into glycosaminoglycan GAG-based systems has accelerated the shift toward personalized medicine in oral mucosal drug delivery. These platforms aim to adapt drug release in response to local physiological cues—such as pH, enzymatic activity, redox state, or hydration—while also offering design flexibility for patient-specific anatomy, dosage, or treatment duration [44,182].

Stimuli-responsive GAG hydrogels and nanogels represent a cornerstone of this approach. For example, thiolated hyaluronic acid (HA–SH) formulations form disulfide linkages with mucins, enhancing mucoadhesion and enabling sustained drug release that is responsive to oxidative degradation in inflamed tissues. These systems have been particularly useful in conditions such as oral ulcers and mucositis, where oxidative stress contributes to pathogenesis and healing dynamics [187].

pH-sensitive platforms have also been developed to release drugs preferentially in the acidic or alkaline microenvironments associated with infection or malignancy. In one study, HA hydrogels incorporating pH-labile crosslinks remained stable at neutral pH but rapidly disassembled in mildly alkaline environments, as found in dental plaque or infected wounds—releasing both antimicrobial agents and anti-inflammatory ions (e.g., Zr^4+^) on demand [264].

In parallel, 3D printing technologies are enabling the fabrication of anatomically tailored mucoadhesive devices. Multi-layer scaffolds incorporating HA and other GAGs can be printed to match lesion shape and depth, optimizing surface contact and controlling drug release gradients. A 3D-printed HA-gelatin-alginate patch was tested in several studies with distinct adhesive and drug-loaded layers that released corticosteroids over four days, offering a proof-of-concept for lesion-specific treatment in chronic oral conditions [285,286,287].

Moreover, multifunctional GAG-based formulations are being designed to address complex pathologies that involve both microbial dysbiosis and inflammation. For instance, a eutectogel composed of HA and xanthan gum was recently formulated with ibuprofen and antimicrobial agents to treat oral lichen planus. This system combined prolonged release (up to 24 h) with broad-spectrum antibacterial activity, reducing both inflammation and pathogen load using a lower drug dose than conventional gels [288].

The development of these personalized and responsive systems aligns closely with the broader trend toward precision therapy. GAG-based carriers can be tuned in terms of molecular weight, crosslink density, and surface functionalization to match patient-specific mucosal conditions or pharmacokinetic requirements. In the future, integration with salivary diagnostics or microbiosensor feedback loops may allow real-time control over drug release, further individualizing care.

In summary, personalization and smart-release features are transforming GAG-based delivery platforms from passive carriers into interactive therapeutic systems. These innovations promise to enhance clinical outcomes, reduce dosing frequency, and improve patient adherence in the management of both acute and chronic oral diseases.

### 9.4. Ongoing Clinical Trials and Translational Challenges

The past five years have witnessed encouraging progress in the clinical development of GAG-based drug delivery systems for the oral mucosa. While several formulations have advanced from laboratory prototypes to human trials, the path toward widespread clinical adoption is still marked by regulatory, manufacturing, and patient-centered challenges.

Multiple clinical trials have evaluated the therapeutic efficacy of HA-based gels and sprays in oral mucositis, aphthous ulcers, and chemotherapy-induced lesions. For instance, a completed Phase IV pediatric study (NCT05818007) assessed a topical HA formulation for oral mucositis in children undergoing chemotherapy. The results suggested modest but statistically significant improvements in lesion healing and patient comfort when used alongside standard oral care [288]. Similarly, HA mouthwashes and bioadhesive gels have been tested in adults with radiation-induced mucositis, showing reductions in pain severity and lesion duration [44].

More advanced GAG-conjugated systems, including HA–drug complexes and HA-coated nanoparticles, are under investigation for oncologic and immunologic applications. ONCOFID-P, a paclitaxel–HA conjugate, has reached Phase III evaluation for localized chemotherapy, although its initial application has been in bladder cancer. However, its design principles—exploiting CD44 targeting and mucosal retention—are highly relevant for oral squamous cell carcinoma and may inform future intraoral formulations [289].

Despite these advances, several translational hurdles remain. First, natural variability in GAG source materials—such as differences in molecular weight, degree of acetylation, and purity—can introduce batch-to-batch inconsistencies, complicating manufacturing and regulatory approval. Moreover, many GAGs (e.g., HA and CS) are hygroscopic, posing stability concerns during storage, particularly in humid environments. To address this, formulations must incorporate protective packaging or use stabilizing additives, such as sugars, amino acids, or crosslinking agents [43].

Scalability is another constraint. Advanced fabrication methods—such as freeze-drying, layer-by-layer deposition, or 3D bioprinting—often require specialized equipment and quality control protocols that are not yet standardized across pharmaceutical manufacturing pipelines. This can lead to high production costs and limit commercial viability unless these processes are simplified or automated [1].

Patient compliance and sensory acceptability also warrant close attention. Mucoadhesive patches and gels must be discreet, palatable, and non-irritating. Overly strong adhesion may cause discomfort or impede natural oral functions, while insufficient adhesion risks premature dislodgement. Moreover, some natural GAGs may trap food debris or interfere with the oral microbiome if used chronically, raising concerns about long-term mucosal compatibility [99].

From a regulatory perspective, there remains ambiguity around whether GAG-based systems are classified as drugs, devices, or combination products. While some HA oral formulations are currently marketed as Class I medical devices or nutraceuticals, more advanced drug-loaded systems will require robust clinical trials demonstrating superiority over standard therapies. This includes head-to-head comparisons with existing gels, rinses, or systemic medications in terms of efficacy, safety, and patient-reported outcomes [36,290,291,292,293,294,295] (Table 6).

Finally, health economic considerations are increasingly important. High-purity GAGs and sophisticated delivery platforms can increase treatment costs, which may limit accessibility unless reimbursement strategies or cost-effective production methods are developed.

## 10. Conclusions

Glycosaminoglycan-based (GAG) platforms have progressed from ancillary excipients to tunable matrices that extend residence, regulate interfacial hydration/charge, and enable controlled, site-directed release on the oral mucosa. Hyaluronan, chitosan (including thiolated derivatives), and alginate now underpin thin films, wafers, hydrogels, and electrospun scaffolds with reproducible gains in mucoadhesion and local pharmacodynamic exposure. Nevertheless, evidence for the most ambitious functions—multi-drug sequencing, receptor-mediated targeting, and light-triggered setting—remains predominantly preclinical, and superiority to standard care has not yet been demonstrated in controlled human studies.

Translation is limited by non-harmonized mucoadhesion/permeation assays and weak in vitro–in vivo correlation; polymer heterogeneity, sterilization-induced molecular-weight drift, and long-term stability; combination-product classification and PMOA justification; and patient-centric usability. Manufacturing risks include content uniformity across large sheets, residual solvents, and sterilization that preserves mechanics and adhesion, necessitating QbD/DoE and inline QA.

Near-term opportunities (12–24 months) include single-drug HA or chitosan films/wafers for clearly defined lesions, xerostomia coatings guided by mucin-synergism metrics, and pilot randomized trials built on standardized panels (ΔG′ with mucin, wet-peel/tensile, TEER-recovery). Longer-term goals (≥3–5 years) involve clinically validated multi-drug and HA–CD44-targeted systems, safe triggerable formats, and selected systemic delivery once human PK/PD is established. Priorities are interlaboratory ring trials, explicit IVIVC development, head-to-head clinical testing versus standard care, post-sterilization re-qualification of properties, and early regulatory and health-economic planning.

## Figures and Tables

**Table 1:** Materials and typical ratios used in oromucosal MDDS (starting-point ranges) (an original table based on the current literature data).

Dosage Form	Film-Forming/Matrix Polymer(s)	Plasticizer(s)	Mucoadhesive Agent(s)/Modifiers	Backing/Release-Direction Control	Other Key Excipients	Typical Ratios (*w*/*w* Unless Noted)
Mucoadhesive films/wafers	HPMC, PVA, pullulan, chitosan, alginate, HA (single or blends)	Glycerol, PEG-400, sorbitol	Carbomer/polycarbophil; chitosan or thiolated chitosan; Ca^2+^ (alginate); catechol grafts (wet adhesion)	Ethyl cellulose; Eudragit RS/RL (laminated/back-coated)	Sweeteners/flavors (min), salivary buffers	Casting solution solids 2–6%; polymer:plasticizer 100:10–30; mucoadhesive co-polymer 10–50% of total polymer; crosslinker (e.g., CaCl_2_) 0.5–2% vs. polymer; API 1–20% of dry film
Buccal/sublingual tablets and lozenges	HPMC (K4M–K100M), NaCMC, MCC (filler), mannitol/isomalt (lozenges)	(Usually none; mouthfeel humectants optional)	Carbopol 934P/polycarbophil; chitosan (1–5%)	Optional impermeable coat (ethyl cellulose/Eudragit) for unidirectional release	Lubricant (Mg stearate 0.5–1%), flavors/sweeteners	Matrix polymer 20–40%; mucoadhesive 1–10%; diluent 20–60%; coat weight gain 2–5%
Gels/creams (semisolids)	Carbomer 940/974; chitosan (acetate); HA; poloxamer 407/188; alginate/pectin blends	Glycerol/sorbitol (humectants)	Polycarbophil/carbomer; chitosan; xanthan/CMC for tack	—	Preservative system; buffer (pH 6–7)	Carbomer 0.2–1.0% (neutralized); chitosan 0.5–2% *w*/*v*; HA 0.1–1%; poloxamer 407 15–22% (+188 1–5%); humectant 5–15%
Sprays (solution/low-viscosity gels)	HA 0.1–0.5%, xanthan 0.1–0.3%, chitosan 0.1–0.5%	— (viscosity kept low for sprayability)	Chitosan/polycarbophil (low %) for mucosal wetting/retention	—	Surfactant (polysorbate 0.01–0.1%), preservative, buffer; target η 10–100 mPa·s	Polymer 0.1–0.5% (total); verify delivered-dose uniformity and droplet size

**Table 2:** Key Properties of major glycosaminoglycans (GAGs) for oral drug delivery (an original table based on the current literature data).

GAG Type	Mucoadhesive Potential	Biodegradability	Bioactivity	Primary Applications
Hyaluronic Acid	High	Yes	Hydration, tissue regeneration, anti-inflammatory	Wound healing, mucoadhesive films
Chitosan	Very High	Yes	Antimicrobial, antioxidant, immunomodulatory	Nanoparticles, films, wafers
Dextran	Moderate	Yes	Tumor targeting, antioxidant	Gels, micelles, nano/micro-particles
Alginate	High	Yes	Mucoadhesion, pH-responsive swelling	Hydrogels, hybrid nanoparticles, wound dressings

**Table 3:** Critical comparison of GAG polymers for oral mucosal drug delivery (an original table based on the current literature data).

Polymer	Charge and Mucoadhesion (Mechanism)	Permeation Effect	Enzymatic and Chemical Stability	Filmability/Comfort	Sterilization Notes	Clinical Signal on Oral Mucosa
Chitosan (incl. thiolated)	Cationic; electrostatic binding to mucins; thiols add covalent disulfides → strong wet adhesion	Reversible TJ opening; report TEER recovery.	Degraded by lysozyme; crosslinking ↑ resistance.	Cast films and fibers; good strength in thin formats.	γ-irradiation can depolymerize (dose-dependent) → verify MW post-sterilization.	RCT in RAS: chitosan film accelerated ulcer size reduction vs. control; well tolerated.
Hyaluronic acid (HA)	Neutral/anionically behaving; H-bonding and hydration lubrication; CD44 binding supports localization in inflamed/tumor tissue.	No intrinsic TJ opening; leverages retention/targeting.	Susceptible to hyaluronidase in saliva; crosslinking or higher MW helps.	Comfortable gels/films; barrier mouthwashes widely used.	γ-sterilization reduces MW; validate properties post-process.	Clinical: HA gels/films/mouthwashes improve pain/healing in aphthae; adjunct in mucositis.
Alginate	Anionic; ionic-gelation mucoadhesion; strength tunable by G/M ratio & Ca^2+^ crosslinking.	No TJ opening; can host nanoparticles for flux.	Stable as Ca-gel; sensitive to ionic strength.	Thin films feasible; taste neutral; good backing layers.	Steam/γ acceptable with property checks (crosslinked forms).	Preclinical antifungal and model drugs in buccal films; limited direct clinical film data in mouth lesions.
Dextran/Dextran sulfate	Neutral/strong anion (sulfated); forms PECs with chitosan (↑ mucoadhesion)	As PECs, may modulate paracellular transport; otherwise minimal intrinsic effect.	Generally enzymatically stable in saliva; sulfated forms anticoagulant.	Films and nanocarriers reported; mouthfeel acceptable.	Depolymerization under harsh radiation possible—verify.	No robust clinical oral-lesion data; platform largely preclinical.
Chondroitin sulfate (CS)	Anionic; hydration/barrier effects; soothing “biolubricant.”	None intrinsic; functions mainly as protective matrix.	Enzymatically degradable (chondroitinases) but generally stable enough in short wear.	Comfortable gels/liners; often combined with HA.	Standard sterilization applicable with QC. (device literature)	Clinical nearby field (esophagus) device evidence with HA+CS barriers; oral-lesion data mostly preclinical/observational.
Dermatan sulfate/Heparin	Strongly anionic; bioactive (anticoagulant/anti-inflammatory).	None intrinsic for permeability; safety caution for bleeding.	Enzymatically degradable; systemic effects possible.	Limited as films; niche.	Standard sterilization significantly degrades the structure and anticoagulant activity of heparin.	Not proven.

**Table 4:** Glycosaminoglycans (GAG)-Based Drug Delivery Platforms for the Oral Mucosa (an original table based on the current literature data).

Platform Type	Composition (Example GAGs)	Advantages	Application Examples
Mucoadhesive Films	Chitosan, hyaluronic acid	Improved residence time, easy application	Aphthous ulcers, sublingual antihypertensives
Hydrogels	HA, chondroitin sulfate	Stimuli-responsive release, high hydration	Periodontitis, oral cancer
Nanoparticles (NPs)	HA-coated NPs, chitosan-based	Targeted delivery, high surface-to-volume ratio	Oral candidiasis, peptide delivery
Textile-Based Scaffolds	Alginate/chitosan woven fibers	Mechanical strength, regenerative potential, conformability	Oral wound healing, post-extraction sockets (dry socket)

**Table 5:** Representative rheology studies relevant to oromucosal GAG systems (an original table based on the current literature data).

Polymer System and Characterization	Equipment/Model	Geometry and Temperature	Study Type	Key Parameters Reported	Principal Results (Brief)	Barrier/Performance Relevance
Sodium chondroitin sulfate + xyloglucan mucoadhesive oral liquid (AL2106); viscosity-matched placebo comparators	Rotational rheometer (model not specified); cone–plate used in all tests	Cone–plate, Ø50 mm; 37 °C; solvent trap/seal to limit evaporation	Flow curves; amplitude sweep (G′/G″); mucin rheological synergism (mixture vs. components)	η($\gamma^{\cdot }$); G′, G″; tan δ; synergism index (Δη)	Positive mucin synergy and elastic reinforcement vs. components; barrier effect confirmed on reconstructed epithelium; methods explicitly detail cone-plate at 37 °C and amplitude sweep parameters.	Directly links rheology to mucoadhesion and epithelial protection—useful benchmark for oral liquids and mouth-coating products.
Hyaluronic acid (HA) and benzyl-ester HA (Hydeal-D^®^) blend (HydC); 1.8% HA + 0.2% Hydeal-D	Anton Paar MCR-102	Cone/plate C60/1 (Ø 60 mm; 1°); 37 °C	Shear-rate ramps; mucin rheological synergism (8% porcine gastric mucin); “washability” on ex vivo mucosa	Apparent η; Δη at 10–150 s^−1^; % formulation retained after washes	HA and HydC show positive Δη; Hydeal-D alone ~0; ~70% of GAGs retained after 3 washes (mucosa model). Detailed instrument and geometry reported.	Provides a reproducible synergy protocol (mucin %, shear window) and a practical retention metric connecting rheology → residence.
HA/Chitosan (CHI) complex coacervates; multiple HA and CHI MW, variable CHI acetylation	Anton Paar MCR-300 with Peltier and solvent trap	Parallel plate (25 mm) or cone-plate (25 mm, 2°); 25 °C	SAOS: strain sweep → frequency sweep; shear-rate sweep; time-sweep for catechol-crosslinking	G′, G″, crossover ω; complex viscosity vs. G* (Winter plot); relaxation time	Coacervates exhibit G′ > G″ and much higher moduli than HA or CHI gels alone (e.g., G′ ≈ 1100 Pa at 10 rad s^−1^ for HA750k/CHI vs. ~20 Pa HA gel); MW and acetylation strongly tune viscoelasticity.	Mechanistic map for tuning film/gel stiffness (comfort vs. retention) and handling (shear-thinning for in-mouth spread).
Chitosan:TPP nanoparticles + reconstituted mucus (tribo-rheology study)	TA Instruments DHR-3	Cone-plate 60 mm, 0.9969°; Peltier 37 °C; plate–plate 40 mm for tribology	SAOS (ω-sweep); flow curves; mucin synergy; triborheology (μ vs. ω under 1 N normal force)	G′, G″; η($\gamma^{\cdot }$); Δη; friction coefficient μ(ω)	Demonstrates pH-dependent viscoelastic and friction changes when nanoparticles interact with mucus; full fixture and temperature reporting.	Adds friction/lubrication readout to standard rheology—useful for xerostomia coatings and comfort claims.
HA solutions (oral context); comparison to human saliva	Rotational rheometry (details in full text)	Not stated in abstract; physiological temperature used in assays	Flow curves; wettability and enzyme compatibility alongside rheology	η and viscoelasticity vs. saliva; contact angles; no major interference with salivary enzymes	HA shows saliva-like viscoelasticity, supporting its role in oral substitutes/liners; complements mucin synergy work above.	Corroborates biorelevance of HA rheology for oral lubrication and film-forming behavior.
Chitosan HCl–mucin mixtures; concentration and polymer/mucin ratio study	Viscometry (capillary/rotational; see article)	Aqueous vs. 0.1 M HCl; ambient	Viscosimetric mucoadhesion (Cheng–Evans modeling); synergy parameter	η_0_ and η∞ from model fits; synergism vs. composition	Identifies two regimes: a minimum-viscosity region and a positive synergy region (excess mucin) that correlates with stronger mucoadhesive joint (also supported by tensile tests).	Classical, easy-to-replicate screening for chitosan–mucin interactions; clarifies why results vary with mucin grade/ratio.
Alginate-containing gels (alginate–gelatin PECs); broad compositional sweep	(Model in paper; MDPI study)	Parallel or cone–plate; temperature ramps	SAOS; creep/recovery; gelation kinetics	G′/G″ vs. T, composition; gel strength thresholds	Defines composition windows that maximize modulus/gel strength in alginate-rich PECs—parameters handy for mucoadhesive scaffold stiffness targets.	Provides design rules for scaffold stiffness → balance between mechanical robustness and oral comfort; relevant for tailoring gels that resist salivary shear but remain patient-tolerable.

**Table 6:** Marketed oromucosal GAG-based mucoadhesive drug-delivery systems (MDDS): composition, active ingredient, indication, and manufacturing notes (an original table based on the current literature data).

Brand (Company)	GAG Component	Active Drug (If Any)	Dosage Form	Primary Indication(s)	Manufacturing/Notes
Gengigel (Ricerfarma)	Sodium hyaluronate 0.2%	None (medical device)	Gel, mouthrinse	Gingivitis/periodontitis adjunct; aphthous lesions; post-surgical care	Semisolid gel or aqueous solution prepared by HA hydration and blending; specific unit operations not publicly disclosed. Clinical use reported in periodontitis/gingivitis studies.
Aloclair Plus (Alliance Pharma)	Sodium hyaluronate (high MW)	None (medical device)	Mouthwash, gel, spray	Aphthous ulcers, minor oral lesions, mucositis symptoms	Solution/gel manufacturing typical for HA devices; method not detailed in public leaflets. Non-inferiority/pragmatic clinical data exist for HA mouthwash/gel in RAS.
Mucosamin (TRB Chemedica)	Sodium hyaluronate + amino acids	None (medical device)	Spray, gel, mouthwash	Prevention/management of chemotherapy/radiotherapy-induced oral mucositis; symptomatic relief	Aqueous HA + amino-acid formulations; pump spray or gel; process not publicly disclosed. Recent randomized and prospective studies report benefit in OM.
Curasept ADS Hyalu Pro Gel (Curasept)	Hyaluronic acid	Chlorhexidine (0.5%)	Mucoadhesive gel	Post-surgical periodontal care; implant surgery; extractions (anti-plaque with mucosal repair support)	Gel blending; marketed with ADS anti-discoloration system. Ongoing/real-world and clinical investigations evaluate HA + CHX combinations.
Chitosan Oral Ulcer Relief Gel (Honest International; ARTG listing)	Chitosan (poliglusam)	None stated	Gel	Oral ulcers and minor lesions (device/medicine per local register)	Registered product in Australia; formulation described as mucoadhesive oral gel; manufacturing specifics not public.

## Data Availability

Not applicable.

## Abbreviations

The following abbreviations are used in this manuscript:

Abbreviation	Full Term
DDS	Drug Delivery System(s)
GAGs	Glycosaminoglycans
HA	Hyaluronic Acid
CS	Chondroitin Sulfate
DS	Dermatan Sulfate
ECM	Extracellular Matrix
CD44	Cluster of Differentiation 44 (cell surface receptor)
HPMC	Hydroxypropyl Methylcellulose
PEG	Polyethylene Glycol
FTIR	Fourier Transform Infrared Spectroscopy
NMR	Nuclear Magnetic Resonance
MS	Mass Spectrometry
LC-MS	Liquid Chromatography–Mass Spectrometry
RPIP-HPLC	Reverse-Phase Ion-Pair High-Performance Liquid Chromatography
RPIP-UPLC-MS	Reverse-Phase Ion-Pair Ultra-Performance Liquid Chromatography–Mass Spectrometry
ESI-MS	Electrospray Ionization Mass Spectrometry
MALDI	Matrix-Assisted Laser Desorption/Ionization
TOF	Time-of-Flight
CE	Capillary Electrophoresis
FACE	Fluorophore-Assisted Carbohydrate Electrophoresis
LIF	Laser-Induced Fluorescence
MTT	Methyl Thiazolyl Tetrazolium (Assay)
LDH	Lactate Dehydrogenase (Assay)
UV-Vis	Ultraviolet–Visible (Spectroscopy)
AFM	Atomic Force Microscopy
SEM	Scanning Electron Microscopy
TEM	Transmission Electron Microscopy
SEM-EDX	Scanning Electron Microscopy with Energy Dispersive X-ray Spectroscopy
PGA	Polyglycolic Acid
PVP	Polyvinylpyrrolidone
PNIPAM	Poly(N-isopropylacrylamide)
PLGA	Poly(lactic-co-glycolic acid)
NP(s)	Nanoparticle(s)
MP(s)	Microparticle(s)
ROS	Reactive Oxygen Species
AMAC	2-Aminoacridone (used in carbohydrate labeling)
HPLC	High-Performance Liquid Chromatography
UPLC	Ultra-Performance Liquid Chromatography
